# Baldur: Bayesian Hierarchical Modeling for Label-Free Proteomics with Gamma Regressing Mean-Variance Trends

**DOI:** 10.1016/j.mcpro.2023.100658

**Published:** 2023-10-07

**Authors:** Philip Berg, George Popescu

**Affiliations:** 1 Institute for Genomics, Biocomputing & Biotechnology, Mississippi State University, Mississippi State, Mississippi, USA; 2 Department of Biochemistry, Molecular Biology, Entomology and Plant Pathology, Mississippi State University, Mississippi State, Mississippi, USA

**Keywords:** proteomics, Bayesian hierarchical model, empirical Bayes, mean-variance trends, latent gamma mixture regression, uncertainty modeling, label-free quantification, data-independent acquisition

## Abstract

Label-free proteomics is a fast-growing methodology to infer abundances in mass spectrometry proteomics. Extensive research has focused on spectral quantification and peptide identification. However, research toward modeling and understanding quantitative proteomics data is scarce. Here we propose a Bayesian hierarchical decision model (Baldur) to test for differences in means between conditions for proteins, peptides, and post-translational modifications. We developed a Bayesian regression model to characterize local mean-variance trends in data, to estimate measurement uncertainty and hyperparameters for the decision model. A key contribution is the development of a new gamma regression model that describes the mean-variance dependency as a mixture of a common and a latent trend—allowing for localized trend estimates. We then evaluate the performance of Baldur, limma-trend, and *t* test on six benchmark datasets: five total proteomics and one post-translational modification dataset. We find that Baldur drastically improves the decision in noisier post-translational modification data over limma-trend and *t* test. In addition, we see significant improvements using Baldur over the other methods in the total proteomics datasets. Finally, we analyzed Baldur’s performance when increasing the number of replicates and found that the method always increases precision with sample size, while showing robust control of the false positive rate. We conclude that our model vastly improves over popular data analysis methods (limma-trend and *t* test) in several spike-in datasets by achieving a high true positive detection rate, while greatly reducing the false-positive rate.

## Highlights

• We propose a Bayesian Hierarchical Model for label-free proteomics data analysis.
• Mean-variance dependencies are described with a new latent gamma mixture regression.
• We evaluate Baldur performance on six label-free proteomics benchmark datasets.
• Our Bayesian method has a much higher precision than limma-trend on all datasets.
• Baldus is available as an R package through CRAN.

## Graphical Abstract

**Figure:**
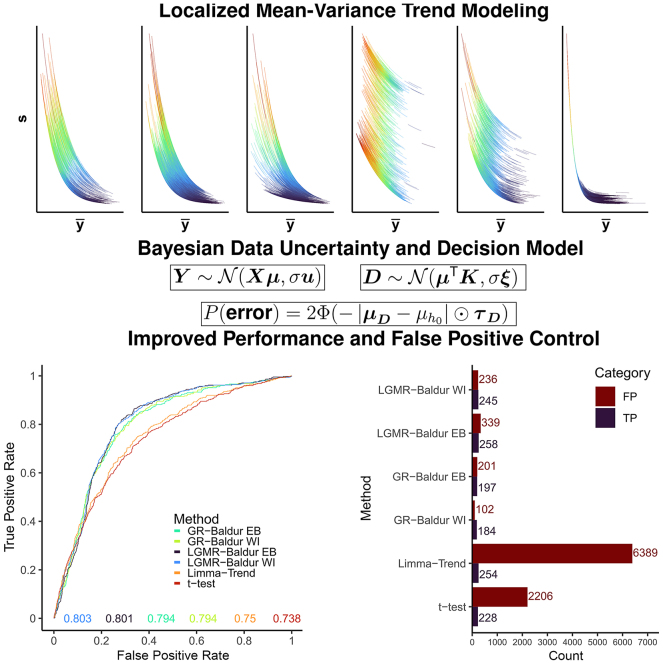

## In Brief

We propose a Bayesian hierarchical decision model to test for differences in means between conditions for proteins, peptides, and posttranslation modifications in label-free proteomics analysis. We introduce a novel Bayesian regression model to characterize local mean-variance trends in the data to estimate measurement uncertainty and hyperparameters for the decision model. Our modeling framework vastly improves precision over limma-trend and *t* test in several spike-in benchmark datasets by achieving a high true positive detection rate while greatly reducing the false positives.

Label-free proteomics is a fast-growing methodology to infer abundances in mass spectrometry proteomics (1, 2, 3). While a common issue in label-free proteomics data is missing values (outside of the scope of this paper), it also tends to produce noisier data than labeling-based methods (4). Extensive research has focused on spectral quantification (2, 5, 6, 7, 8, 9, 10) and peptide identification (9, 10, 11). However, research toward modeling and understanding the end product of quantitative proteomics data and how to utilize dataset level information in statistical testing for differences in means is scarce. Mainly ANOVA or *t* test methods are applied for this analysis (9, 11, 12, 13, 14), but some work toward using mixed effect-, regression-models, and Stouffer’s method, have been developed for total proteomics analysis (15, 16, 17, 18). Still, none of them can deal with generic datasets (DSs), for example, peptidomics, phosphoproteomics, etc. Therefore, our focus here is on the statistical analysis of differences in mean abundances of peptides (or proteins, post-translational modifications, etc.) between different conditions (WT/mutant, control/treatment, time series, etc.). To this end, we present a Bayesian decision method (Baldur) that uses gamma regression (GR) to estimate hyperparameters according to the mean-variance (M-V) trend and the uncertainty of individual measurements. In particular, we propose a new method for modeling the variance component using a gamma distribution. In addition, we develop an improved GR model that describes the M-V dependency as a mixture of a common and a latent trend—allowing for localized estimates of the M-V trend. This is then used for inference of measurements' uncertainty and hyperparameters for the variance prior. We then evaluate the performance of Baldur, *t-*test, and limma-trend on five total proteomics DSs and one post-translational modification (PTM) DS. Importantly, we find that Baldur drastically improves the decision in PTM data (19) over limma-trend and *t* test. Likewise, we see significant improvements using Baldur over the other methods at small spike-in quantities in one of the total proteomics DSs (14). Further, we show that Baldur improves the performance on the remaining five total proteomics DSs (19, 20, 21, 22) over all other models by reducing the number of false positives. Lastly, we performed empirical power analysis to analyze the methods’ precision when increasing the number of replicates. We found that the Baldur methods always gain power with sample size, while showing robust control of the false positive. On the other hand, limma-trend and *t* test increase in power with increased sample size but at the expense of reduced false positive (FP) control leading to decreased precision.

In conclusion, we have developed novel ways of modeling the M-V dependency and a new Bayesian statistical decision method for proteomics. Our Bayesian model is particularly robust for noisy data and shows improved performance over the state-of-the-art decision methods (3, 19, 20) while our novel M-V modeling improves the statistical decision.

## Experimental Procedures

### Datasets

We used six previously published (14, 19, 20, 21, 22) label-free spike-in benchmark DSs to evaluate model behavior and performance. Two of the benchmark DSs published in (19) were produced by label-free quantification, one on total proteomics with the Universal Proteomics Standard Set 1 (UPS1) spiked in at 1:2:4 times concentration in a Chlamydomonas background, and the other is a PTM DS using a reversibly oxidized cysteine enrichment protocol with *Saccharomyces cerevisiae* spiked in at 1:2 concentrations to a Chlamydomonas background (see original paper for details). We refer to these DSs as UPS-DS and Yeast-DS, respectively. The UPS-DS has four replicates per spike-in (condition), and Yeast-DS has three. In addition, we used the data published by Ramus *et al.* (14) that has UPS1 spiked in at nine different concentrations to a yeast background with three replicates in each condition and is also a label-free quantification DS. We also investigated three previously published data-independent acquisition DSs (20, 21, 22). The first DS published by Fröhlich *et al.* (20; called Human-DS from here on) had *Escherichia coli* proteome spiked-in at 6:12:25 concentrations to a background of a heterogeneous human tumor population. Bruderer *et al.* (21) had a stable human cell line (HEK-293 cells) as a background and a complex design of UPS2 spike-in at three different master mixes producing eight different conditions each with three replicates. Finally, Navarro *et al.* (22) had a background of human (cervix carcinoma; HeLa) cells with *E. coli* and yeast (*S. cerevisiae* bayanus strain LALVIN EC-1118) spike-in with ratios 4:1 and 2:1, respectively. Table 1 summarizes the properties of these DSs.

**Table 1:** Properties of the datasets investigated here

Dataset	TP	FP	Total	Conditions	Replicates
Yeast	448	1787	2235	2	3
UPS	392	10,207	10,599	3	4
Ramus	292	4654	4946	9	3
Human	404	2902	3306	3	23
Bruderer	12	3773	3785	8	3
Navarro	3444	3145	6589	2	3

TP (true positives) is the number of spike-in examples, TN (true negatives) the size of the background, Total is the sum of the two, Conditions is the number of different spike-in concentrations, and replicates is the number of Replicates per condition.

### Data Preprocessing

For the Yeast-, UPS-, Ramus-, Navarro-, and Bruderer-DS, normalization was done by calculating the scale factors described in (23), and dividing each sample accordingly. That is, let $y_{ij}$ be the measurement of the *i*:th peptide in the *j*:th sample. The normalization constant $s_{j}$ for the *j*:th sample is then given by Equation 1

(1) $$s_{j}=\underset{i:y_{ij}\in R\forall j}{\text{median}}\frac{y_{ij}}{{\left(\prod_{j=1}^{m}y_{ij}\right)}^{\frac{1}{m}}}$$

The normalized data $y_{ij}^{N}$ was then given by Equation 2.

(2) $$y_{ij}^{N}=\frac{y_{ij}}{s_{j}}$$

Here, $s_{j}$ was calculated using rows without missing values, and after normalization, data was $\log_{2}$-transformed. Lastly, Yeast-, UPS-, Ramus-, Navarro-, and Bruderer-DS were imputed using missForest (24). For the Navarro-DS (22), the data was first processed with the MSstats (25) functions **SpectronauttoMSstatsFormat**, **dataProcess** with the flag normalization set to FALSE, followed by **quantification** and exponentiation ($2^{y_{ij}}$ for measurement $y_{ij}$) after which above procedure was followed. For the Human-DS, columns corresponding to conditions with no spike-in were discarded, then rows with missing values were filtered out before normalization, to follow the arguments presented in the original study (20). For the empirical power analysis, the columns were subset from the normalized data.

### Model Inference

For inference of the latent gamma mixture regression (LGMR) model, we set κ=0.001. Then, we used RStan’s (26) No-U-Turn Sampler using five chains each with 500 warm-up draws, 2000 post warm-up samples (iter set to 2500), adapt_delta set to 0.9 for everything except the empirical power analysis. For the empirical power analysis, we ran 20 chains with 500 warm-ups draws and 2000 post warm-up samples. For the calculation of the normalized root-mean-square error (NRMSE), Equation 15 was calculated during the sampling substituting ${\hat{s}}_{i}$ with $\mu_{i}$. The sample means of the posterior were then used as point estimators and presented in Table 2. Inference of the posterior distribution for the data and decision model was done with RStan’s No-U-Turn Sampler using four chains each drawing 1000 burn-ins and 1000 samples per peptide. The parameters for the GR were estimated using R’s (27, version 4.2.0; https://www.r-project.org/) function **glm** (28), and the shape parameter was estimated using the R package stats’s **summary.glm** function. For integrating D (Equation 10), we used the normal cumulative distribution function as implemented in R’s **pnorm** function using the sample mean and sample SD from the posterior draws.

**Table 2:** Table of inferred regression parameters and normalized root mean squared error for LGMR model

Dataset	α	$\gamma_{0}$	$\gamma_{0L}$	$\gamma_{\hat{y}}$	$\gamma_{\hat{y}L}$	NRMSE
Yeast	2.245	7.482	−1.191	0.874	0.427	0.664
UPS	7.372	6.816	−2.138	0.729	0.451	0.455
Ramus	7.766	7.284	−1.655	0.297	0.547	0.439
Human	17.651	7.171	−1.215	0.25	0.007	0.388
Bruderer	5.215	6.405	−2.596	0.501	0.031	0.692
Navarro	2.916	7.156	−1.759	1.385	0.02	0.693

Numbers represents posterior means.

Abbreviation: NRMSE, normalized root-mean-square error.

### Running Limma and *t* Test

For running limma-trend, we first ran **lmFit**, **contrast.fit**, and then **eBayes** was ran with Robust and trend set to TRUE. The *p*-values were then extracted with **topTable** with adjust.method set to ”fdr” and number set to Inf. For *t* test, we used R’s **t.test** function with var.equal = TRUE, and then ran **p.adjust** with method set to ”fdr” on the *p*-values (within each comparisons).

### Performance Metrics

For performance, all decisions were calculated over the closed set [−0.1,1] so the ROC curves always start at the origin. True positive rate (TPR) was calculated according to Equation 3, false-positive rate (FPR) according to Equation 4, precision according to Equation 5, and Mathew’s correlation coefficient (MCC) according to Equation 6.

(3) $$\text{TPR}=\frac{\text{TP}}{\text{TP}+\text{FN}}$$

(4) $$\text{FPR}=\frac{\text{FP}}{\text{FP}+\text{TN}}$$

(5) $$\text{Precision}=\frac{\text{TP}}{\text{TP}+\text{FP}}$$

TP=True PositiveTN=True Negative

FP=False PositiveFN=False Negative

(6) $$\text{MCC}=\frac{\text{TP TN}-\text{FP FN}}{\sqrt{\left(\text{TP}+\text{FN}\right)\left(\text{TP}+\text{FP}\right)\left(\text{TN}+\text{FN}\right)\left(\text{TN}+\text{FP}\right)}}$$

### Empirical Time Complexity Analysis

For the evaluation of model time complexity, we used the microbenchmark package in R. For the data and decision model, we used the empirical Bayes (EB) prior, and the GR model was used to infer model uncertainties and hyperparameters before starting the evaluation. For the LGMR model, we used five chains and five parallel workers for each run using 500 warm-ups and 2000 posterior samples. Both evaluations were performed ten times for each DS.

## Results

### Bayesian Data and Decision Model

Here, we describe a Bayesian hierarchical model (Baldur) to analyze differences in peptide (or protein, PTM, etc., but for simplicity, we will use peptide from here on) abundances. This model will be applied to each peptide independently, and as such, we will describe it from the perspective of one single peptide. We assume the peptide’s data to be normally distributed with *C* different conditions (control/treatment, time points, etc.) each with $n_{c}$ measurements. Then, we assume that the measurements within the *c*:th condition have a common mean $\mu_{c}$. We model each peptide’s data with a common SD σ (unique to each peptide) and a measurement-specific uncertainty, $u_{i}$ for the *i*:th observation, as a multiplicative factor that describes its change of variance from σ. Hence, the uncertainty is a correction for the unobserved measurement-specific variance around the mean. Further, we assume that all measurements and means in each condition are independent and therefore have zero covariance. We then model the means with a group-level effect (29) and assume it is proportional to σ. We use the expanded noncentered parameterization which has been shown to increase sampling convergence and efficiency, and allows for increased model flexibility (29, 30, 31, 32, 33, 34, 35). This allows the model to adjust the posterior variance of each $\mu_{c}$, while still being constrained on σ and to shift the mean proportionally to σ. Finally, σ is assumed to be a gamma random variable with shape and rate parameterization, with hyperparameters estimated from the M-V trend. The data model is summarized in Equation 7.

(7) $$\begin{array}{c} Y∼N\left(X\mu ,\sigma u\right),\;\mu ∼N\left(\mu_{0}+\eta \sigma ,\sigma \right)\\ \sigma ∼\Gamma \left(\alpha ,\beta \right),\;\eta ∼N\left(0,1\right) \end{array}$$

Here, Y is a column vector of N observations, μ is a column vector of the C means, X is an N-by-C design matrix, $\sigma^{2}$ is the common variance, u is a column vector of the uncertainties, and η is a column vector (of length *C*) for group-level effects.

Baldur has two prior choices for $\mu_{0}$, one EB prior, and one weakly informative (WI) prior. The EB prior assumes a normal prior on $\mu_{0}$ similar to the normal–normal compound model in (36, 37). The EB prior assumes that the mean of $\mu_{0}$ is the sample mean with the variation set as twice the common variance normalized by the number of measurements (Equation 8). Here, $\bar{y}$ is a column vector of the sample means in the C conditions, and $n_{R}$ is a column vector of the scaling constants in the C conditions. The WI prior uses a normal distribution with a large variance (Equation 9).

(8) $$\mu_{0}∼N\left(\bar{y},\sigma n_{R}\right),n_{R}={\left[\frac{\sqrt{2}}{\sqrt{n_{1}}},\frac{\sqrt{2}}{\sqrt{n_{2}}},\ldots ,\frac{\sqrt{2}}{\sqrt{n_{C}}}\right]}^{T}$$

(9) $$\mu_{0}∼N\left(0,10\right)$$

Next, we model our decision statistic D for comparisons of interest as a normal distribution with mean equal to a contrast of interest and variance equal to the common variance ($\sigma^{2}$; Equation 10) normalized by the contrast weighted sample size, ξ.

(10) $$D∼N\left(\mu^{T}K,\sigma \xi \right),\;\xi_{m}=\sqrt{\sum_{i=1}^{C}\frac{\left|k_{im}\right|}{n_{i}}}$$

where K is a C by M contrast matrix (with M contrasts; Equation 11) of interest with the constraint that each column’s positive values sum to one and negative terms sum to minus one.

(11) $$K=\left[\begin{array}{cccc} k_{11} & k_{12} & \ldots & k_{1M}\\ k_{21} & k_{22} & \ldots & k_{2M}\\ \vdots & \vdots & \vdots & \vdots \\ k_{C1} & k_{C2} & \ldots & k_{CM} \end{array}\right]$$

$$\sum_{i=1}^{C}k_{im}=0\;\&\;\sum_{i=1}^{C}\left|k_{im}\right|=2\;\forall m\in \left[1,2,\ldots ,M\right]$$

This allows for pairwise and nonpairwise comparisons, for example, comparing the mean in one condition against the mean of two others.

Finally, we estimate the probability of error by integrating the tails of D. Let Φ be the cumulative distribution function of the standard normal distribution, $\mu_{D}$ the mean(s) of the posterior(s) of D, $\tau_{D}$ the reciprocal of the posterior SDs (square root of the precision). Further, let the null hypothesis be that the difference in means is equal to $\mu_{h_{0}}$. Then the probability of error(s) is then defined according to Equation 12.

(12) $$P\left(\text{error}\right)=2\Phi \left(-\left|\mu_{D}-\mu_{h_{0}}\right|⊙\tau_{D}\right)$$

where ⊙ is the Hadamard product.

### Modeling the Mean-Variance Trend

Here, we will describe EB methods for estimating the hyperparameters of σ. Let $s=\left(s_{i}\right)\in R_{+}^{p}$ be a column vector of the sample SDs, and $\bar{y}=\left({\bar{y}}_{i}\right)\in R^{p}$ of the sample means in the *p* peptides of a DS of interest. Here, we use the sample SD since the data model (Equation 7) assumes that each measurement has a unique variance.

### Gamma Regression for the Mean-Variance Trend

The first inference model uses a GR for estimating model hyperparameters. Let s be gamma-distributed and parameterized as described in Equation 13 (*i.e.*, a GR with log-link function).

(13) $$s∼\Gamma \left(\hat{\alpha },\hat{\beta }\right)\;\hat{\beta }=\frac{\hat{\alpha }}{e^{{\hat{\gamma }}_{0}+{\hat{\gamma }}_{\bar{y}}\bar{y}}}$$

where Γ(.,.) is the gamma distribution with shape, rate parameterization, and ${\hat{\gamma }}_{i}$ s are the inferred regression parameters. We define the uncertainty for some measurement $y_{ij}$ as the expected SD (Equation 14).

(14) $$u_{ij}=E\left[s_{i}|{\hat{\gamma }}_{0},{\hat{\gamma }}_{\bar{y}},y_{ij}\right]=e^{{\hat{\gamma }}_{0}+{\hat{\gamma }}_{\bar{y}}y_{ij}}$$

Supplemental Fig. S1 shows the fitted GRs to the DSs investigated here (see the DSs section in Experimental Procedures for details). We found that the regression model describes the UPS-DS trend well. But, for the other DSs (in particular for the Human-DS), we observe that a single GR model cannot capture the M-V distributions well. From Table 3, we see that the slopes and intercepts have similar values, except for the Human- and Bruderer-DS with smaller intercepts (both) or slope (Human-DS). In addition, the shape parameter is slightly larger for the Human-DS than the other five. We then calculated the NRMSE (38) to determine the goodness-of-fit.

(15) $$NRMSE=\sqrt{\frac{\frac{1}{p}\sum_{i=1}^{p}{\left(s_{i}-{\hat{s}}_{i}\right)}^{2}}{Var\left(s\right)}}$$

where ${\hat{s}}_{i}$ is the predicted SD of the i:th peptide, and Var(.) is the variance. We found that all DSs generated a similar error with the GR model but the Navarro-DS NRMSE was slightly lower.

**Table 3:** Table of inferred regression parameters and normalized root mean squared error for GR model

Dataset	α	${\hat{\gamma }}_{0}$	${\hat{\gamma }}_{\bar{y}}$	NRMSE
Yeast	1.337	1.352	−0.233	0.818
UPS	1.855	0.982	−0.267	0.819
Ramus	1.895	2.719	−0.193	0.837
Human	3.011	0.041	−0.033	0.996
Bruderer	1.635	0.259	−0.151	0.97
Navarro	1.154	1.246	−0.334	0.738

Abbreviation: NRMSE, normalized root-mean-square error.

### Latent Gamma Mixture Regression

To further increase the precision of the M-V trend modeling for each peptide, we propose a LGMR model. We assume that each peptide’s variance is a mixture of a common and a latent trend—allowing for localized estimates of the M-V trend parameters. The model starts with the same formulation as for the GR model ((13), (16)) but with a half-Cauchy as a weak prior on α (Equation 17; 29).

(16) $$s∼\Gamma \left(\alpha ,\beta \right),\;\beta =\frac{\alpha }{\mu }$$

(17) α∼Half−Cauchy(25)

We then assume that the mean of s is a mixture of the two trends with one intercept and one slope each, and the *i*:th peptide has $\theta_{i}\in \left[0,1\right]$ of the latent trend (Equation 18).

(18) $$\mu =\exp\;\left(\gamma_{0}-\gamma_{\bar{y}}f\left(\bar{y}\right)\right)+\kappa \;\exp\;\left(\theta ⊙\left(\gamma_{0L}-\gamma_{\bar{y}L}f\left(\bar{y}\right)\right)\right)f\left(x\right)=\frac{x-\mu_{\bar{y}}}{\sigma_{\bar{y}}}$$

where κ is some small constant (here we will use 0.001) that defines the smallest possible contribution of the latent trend and *f* gives the standardized sample means (with $\mu_{\bar{y}}$ and $\sigma_{\bar{y}}$ being the mean and sd of $\bar{y}$, respectively). We then choose a uniform distribution as an uninformative prior on $\theta_{i}$ (Equation 19).

(19) $$\theta_{i}∼U\left(0,1\right)$$

We set the slope to always be negative by limiting the slope coefficients to positive values ($\gamma_{\bar{y}},\gamma_{\bar{y}L}\in \left[0,\infty \right]$; Equation 18). To this end, we used a half-normal prior on the slope coefficients.

**Figure:**
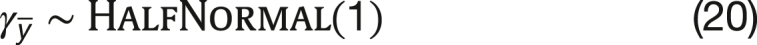

**Figure:**
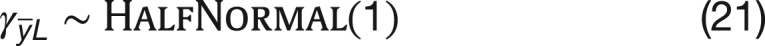

We then set priors on the intercepts. For the common intercept we used a standard normal distribution. For the latent intercept, we set a right-skewed prior using a skewed-normal distribution and setting the α parameter to a large positive value. In addition, we set a large variance by putting a large ω parameter to make the prior weaker. Finally, the location parameter small positive for the latent intercept to force it larger and accommodate for the shrinkage by κ.

(22) $$\gamma_{0}∼N\left(0,1\right)$$

**Figure:**
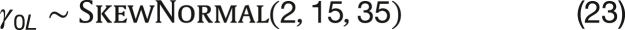

As for the GR model, we define the uncertainty for some observation $y_{ij}$ as its expected sd (Equation 24).

(24) $$u_{ij}=E\left[s_{i}|\theta_{i},\gamma_{0},\gamma_{0L},\gamma_{\bar{y}},\gamma_{\bar{y}L},y_{ij}\right]=\exp\;\left(\gamma_{0}-\gamma_{\bar{y}}f\left(y_{ij}\right)\right)+\kappa \;\exp\;(\theta_{i}\left(\gamma_{0L}-\gamma_{\bar{y}L}f\left(y_{ij}\right)\right)$$

**Fig. 1:**
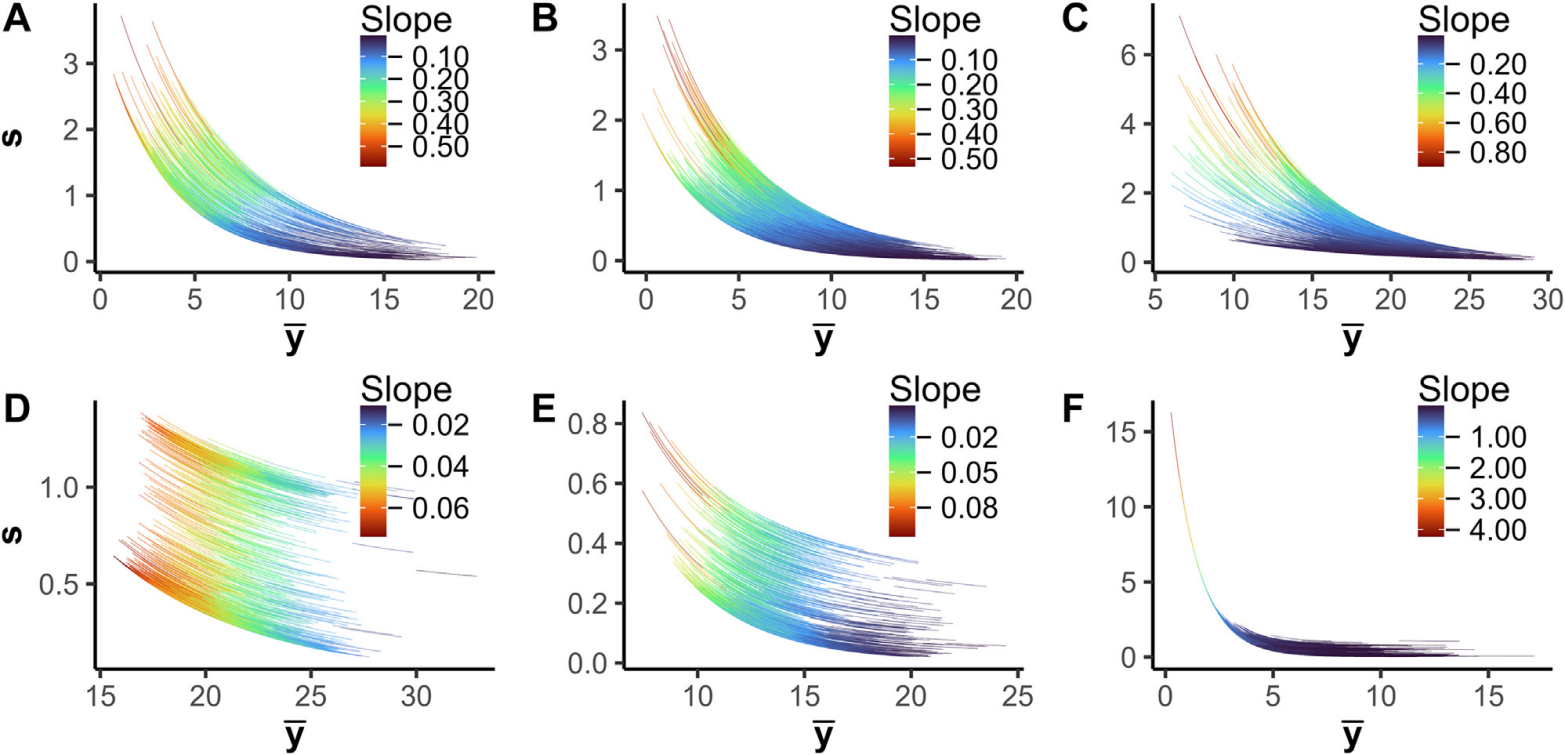
**Themean-variancetrendwith the locally estimated gamma regression.** M-V trends in Yeast-, UPS, Ramus-, Human, Bruderer-, and Navarro-DS (*A*–*F*, respectively), with the *x*-axis showing the sample mean, and the *y*-axis showing the sample SD. Each line indicates the M-V trend of a corresponding peptide, and the color indicates the derivative at the peptide’s mean.

The inferred regression parameters of the LGMR model for the DSs investigated here are shown in Table 2, and the model is visualized in Figure 1. We found that all DSs had unique regression patterns that resemble their corresponding M-V trend (Supplemental Fig. S1). From Table 2, we found that Ramus-DS and UPS-DS had similar α values, while the Yeast and Navarro-DS values were smaller, Bruderer-DS in between the four, and Human-DS was significantly larger; similar to the GR model. We found that the Human-DS had the best fit, followed by the Ramus-, UPS-, Yeast-, Navarro-, and Bruderer-DS. Finally, compared to the GR model, we found that the LGMR model gave a better fit across all DSs (Tables 2 and 3). Taken together, we have developed a new Bayesian regression model using a latent mixture that can describe well the local M-V trend of the DSs investigated here.

### Algorithmic Description

**Table:** 

Algorithm 1 description of Baldur	
Input	
y	Input data
X	Design Matrix
C	Contrast Matrix
Reg	Which regression model to use.
MP	Which means prior to use for the data model
Output	
$y^{out}$ Posterior estimates	
$\left(s,\bar{y}\right)$←CalculateMean-StandarDeviation(y)	
**if** Reg=LGMR **then**	
GamReg ←FitLGMR($s,\bar{y}$)	
else	
GamReg ←FitGR($s,\bar{y}$)	
end **if**	
u←PredictGammaRegression(GamReg, y)	
$\left(\hat{\alpha },\hat{\beta }\right)$←PredictGammaRegression(GamReg, $\bar{y}$)	
**for**i∈{1,2,…,p}**do**	
Posterior ←Sample(MP, $y_{i}$, ${\bar{y}}_{i}$, $\left(\hat{\alpha },{\hat{\beta }}_{i}\right)$, $u_{i}$)	
Estimates_*i*_←SummarizePosterior(Posterior_[σ,μ,D]_)	
**end for**	
$y^{out}$← Estimates	

The procedure for implementing the Bayesian decision is described in algorithm 1. The user needs to input their data, a design matrix, a contrast matrix, a choice of regression model to use, LGMR or GR, and finally a choice of mean prior, EB or WI. Baldur then fits the regression model and uses it to infer uncertainties as well as hyperparameters on σ. It then runs the decision model on each peptide separately and produce a summary statistic of the fit (this allows for a highly efficient parallel computation setup). In particular, Baldur returns the mean, median, a 95% credibile interval, the R-hat, and the efficient sample size for parameters of interest.

### Performance Evaluation

For the performance evaluation, with the exception of the Bruderer-DS, we evaluated DSs on a per-comparison basis; due to the very few true positives (TPs) in the Bruderer-DS (Table 1), we analyzed it over all the $\left(\begin{array}{c} 8\\ 2 \end{array}\right)$ possible comparisons at the same time. In addition, due to the complex mixes of the TPs in the Bruderer-DS, there are no generic ways to define the fold change of the TPs in the different comparisons.

### Receiver Operator Characteristics

To evaluate the performance of the models presented here, we generated receiver operator characteristic (ROC) curves of the six benchmark DSs. We evaluated the following methods: Baldur with both priors and regression models, limma-trend, and *t* test since they are generally used in recent studies (3, 14, 19, 20, 39, 40, 41, 42). For limma-trend and *t* test, we applied false discovery rate correction using the method described in (43).

**Fig. 2:**
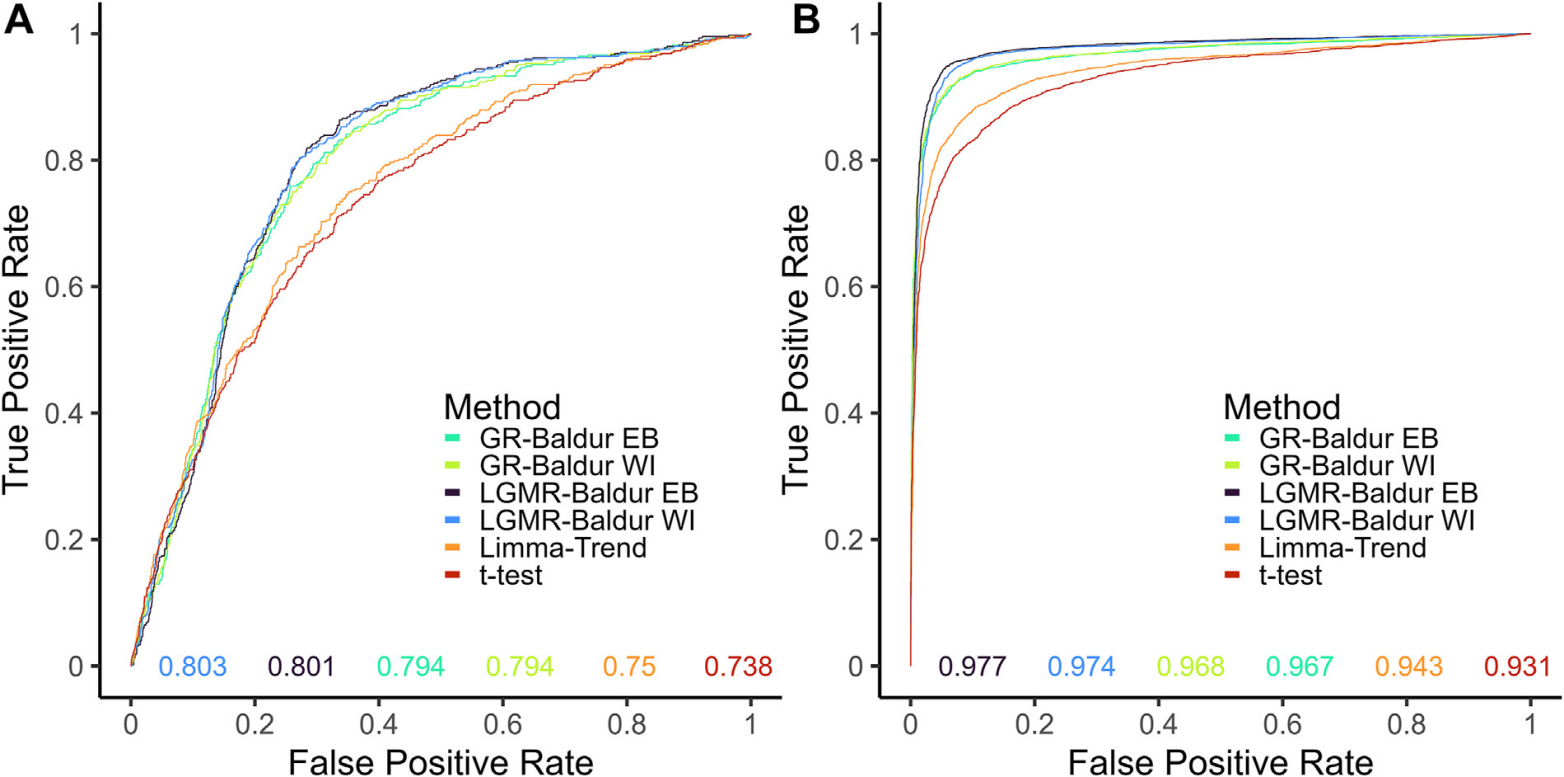
**Receiver operator characteristic curves for theYeast-DSandNavarro-DS.** The Yeast-DS ROC is shown in *A* and the Navarro-DS ROC in *B*. The different colors indicate the evaluated models: LGMR-Baldur is the Baldur method with the LGMR model to estimate σ priors and uncertainty, GR-Baldur uses the GR model to estimate σ priors and uncertainties, EB and WI indicates the empirical Bayes prior or the weakly informative prior on the mean, limma-trend as described in Experimental Procedures, and *t* test is used with pooled variance. The *x*-axis shows the false-positive rate, and the *y*-axis shows the true-positive rate. The *colored numbers* on the bottom of the plots show the area under the curve with colors corresponding to the different models.

For the Yeast-DS (Fig. 2*A*), we observed an increased performance using Baldur. In particular, all Baldur-based models improved on limma-trend and *t* test perfomance. In addition, the LGMR model for parameter inference slightly improved on the GR model, and the WI prior for the mean had a marginally better performance than the EB prior.

For the UPS-DS, we show the ROC curves for both pairwise and nonpairwise comparisons (Supplemental Fig. S2*A*). While all methods generally performed well on the pairwise comparisons, Baldur with the LGMR model has a notably better performance in all comparisons, Baldur with the GR model is second, followed by limma-trend and *t* test. In addition, we found that Baldur-based models had a similar area under the ROC curve (auROC) across all pairwise comparisons, while limma-trend and *t* test had a larger spread in performance for the different comparisons. In addition to pairwise comparisons, we also studied nonpairwise design for the UPS-DS to evaluate the performance of the statistical decision at small and intermediate average log fold changes of the spike-in peptides. We found that, all methods showed similar performance for the larger fold changes, but for the very small fold change of *fmol50 versus fmol25 and fmol100* (*i.e.*, the fold change of TPs are 0.8) we found that LGMR-Baldur methods outperformed GR-Baldur which had a slightly better or equal performance to limma-trend. In addition, the EB prior showed slightly better performance than the WI prior.

For the Human-DS (Supplemental Fig. S2*B*), we found that all methods had similar performance across all comparisons except one (1:12 *versus* 1:6), where LGMR models had slightly better performance.

**Fig. 3:**
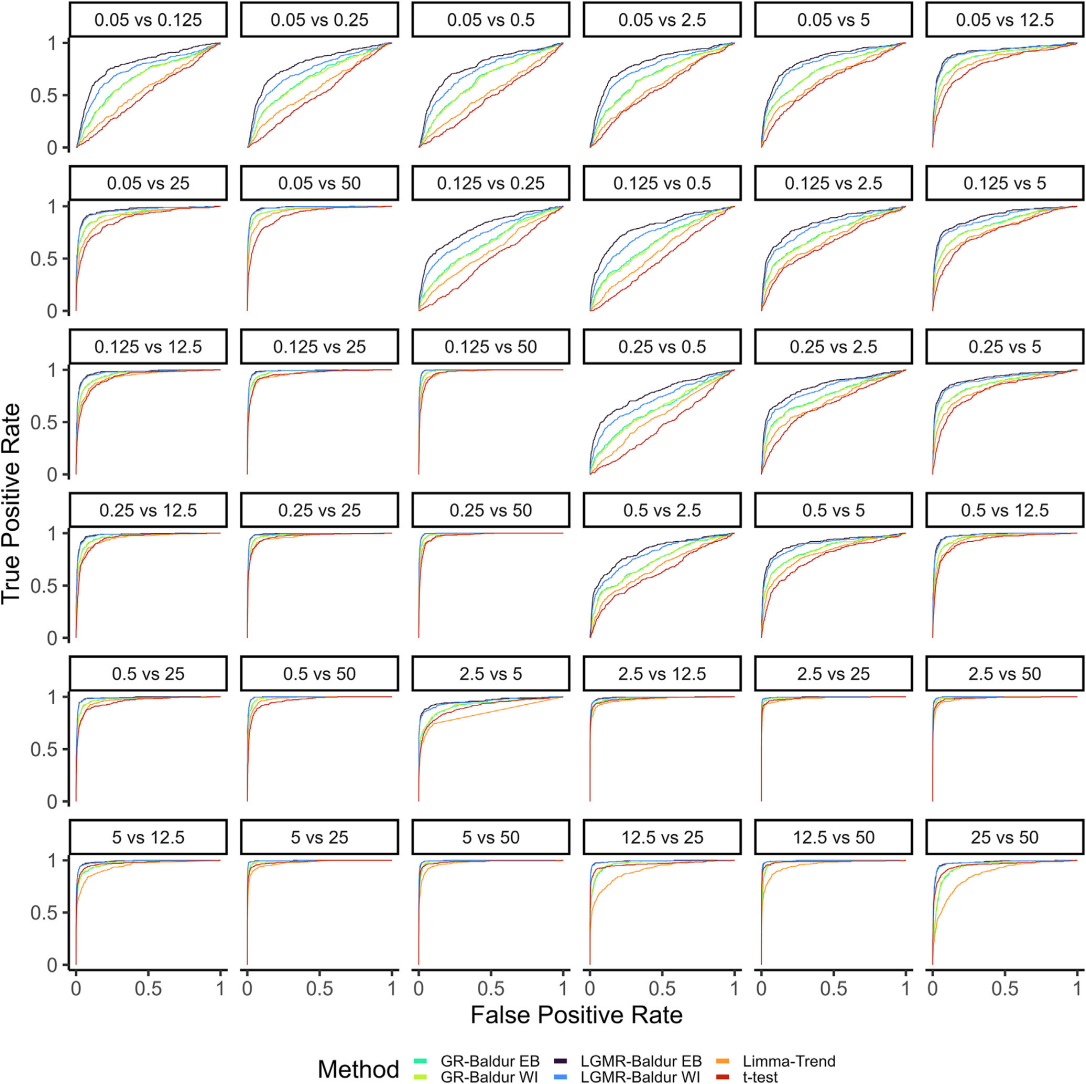
**Receiver operator characteristic curves for the comparisons in the Ramus-DS.***Facet titles* indicate what comparison is being evaluated together with the fmol spike-in concentration of the UPS1 of the two conditions. Color, axis, numbers, and decision as described in Figure 2.

For the Ramus-DS (Fig. 3), we identify a wide range in performance for different comparisons ranging from easy (*e.g.*, 5 *versus* 50) to hard (*e.g.*, 0.125 *versus* 0.5). Still, we found that both GR and LGMR Baldur models can improve performance significantly, when the spike-in quantities are in low concentrations and involve smaller fold changes between conditions. In particular, we see performance improvements for comparisons where the spike-in concentration is lower than 12.5 fmol. Conversely, we find comparable performance for all methods when the fold changes are substantial or when the spike-in concentrations are ample. Still, it is evident that Baldur with the LGRM model performs highest in all 36 comparisons, while GR-based inference is second.

For the Bruderer-DS (Supplemental Fig. S3), we found that the LGMR model drastically outperformed all other models—increasing the auROC by almost 10% over limma-trend and *t* test, and 5% over the GR models. In particular, we observed that the LGMR models attain a higher TPR much faster at lower FPRs than the other models.

For the Navarro-DS (Fig. 2*B*), we see that Baldur methods tend to gain TPR at a faster rate than *t* test and limma-trend. In particular, LGMR inference tends to max out the TPR at an FPR of about 0.1, while GR attains the same TPR around 0.5 FPR, and *t* test at around 0.8 FPR. This indicates the LGMR inference returns TPR at a much lower FPR cost for this DS, followed by GR models, limma-trend and lastly *t* test.

Summarizing, Baldur has drastic performance improvement in noisier conditions of proteomics measurements, with the LGMR as the best-performing alternative and GR second. On the other hand, for less noisy (easier) DSs (or comparisons; *e.g.*, large fold change), Baldur is marginally better than limma-trend or *t* test, as these methods already have large auROC and therefore have little room for improvement. In addition, we observed that the LGMR model consistently improves Baldur’s performance over GR model inference. Finally, we observed that Baldur has dependable performance across all DSs investigated here, while limma-trend and *t* test can show considerable variation in performance between comparisons.

### Decision Patterns

While ROC curves can produce a summary statistic over a classifier’s support—it is inadequate for a deeper understanding of specific statistical thresholds typically used in the significance analysis of proteomics data. Thus, we investigated MCC (44, 45) as a point estimate of statistical decisions over a range of significance levels. The motivation for using MCC is that all DSs (except Navarro-DS) are heavily unbalanced between TPs and true negatives (Table 1), and MCC has, arguably (46, 47), good properties for unbalanced data.

For Yeast-DS (supplemental Fig. S4), we found that LGMR does performed slightly better than GR and the EB is better paired with LGRM-based inference, while WI works better with GR-based parameter estimation. In addition, we found that Baldur methods always have a larger MCC than limma-trend and *t* test. Finally, we found that the Baldur methods make an optimal decision around 1 to 5%, while limma-trend and *t* test peak very close to 0 and subsequently decay with the significance level.

Similarly to the ROC curves, the Ramus-DS showed a wide range of performance for the MCC (Supplemental Fig. S5) between different comparisons. Still, Baldur using GR and LGRM inference tended to produce better-performing decisions that generally decay slower with the significance level than limma-trend and *t* test that often peak at very small significance levels. Importantly, while some ROC curves (Fig. 3) suggested similar performance, the MCC showed substantial differences at typical significance levels. In particular, we see that Baldur-based models showed better performance when compared to limma-trend and *t* test in the 5 *versus* 50 comparisons; all methods showed good ROC performance, while limma-trend and *t* test have significantly decreased MCC. Finally, we see that the Bayesian decision tends to have robust performance, while *t* test and limma-trend have unexpected drops in performance, in particular at lower significance levels.

For the UPS-DS (Supplemental Fig. S6), we again found that Baldur-based decisions tend to outperform limma-trend and *t* test. In all pairwise comparisons, we find that the LGRM model tends to produce the best decisions. In particular, we find that the WI prior slightly increases performance at the comparison *fmol25 versus fmol100* that has the larger fold change. We find that while the GR method is slightly worse than limma-trend for the *fmol50 versus fmol100* and *fmol25 versus fmol50* comparisons it shows better performance in the *fmol25 versus fmol100* comparison. For the nonpairwise contrasts, we found that the LGMR and GR models performed better at typical significance levels. For the most challenging contrast (*fmol50 versus fmol25 and fmol100*), we find a significant drop in Baldur performance, while limma-trend does not produce any decision until a large significance level (greater than 0.19).

For the Human-DS (Supplemental Fig. S7), we observed that LGMR improves the decision over limma-trend and *t* test in all three comparisons, while the GR model has better performance in two comparisons. Surprisingly, Baldur performs considerably better at the largest fold change, where limma-trend and *t* test rapidly drop in performance. In addition, we observe that both priors behave (almost) identically in all three comparisons, likely due to the large number of replicates in this DS.

In the Bruderer-DS (Supplemental Fig. S8), we found that, contrary to the ROC curve (Supplemental Fig. S3), the prior choice had the largest impact on the performance. In particular, the WI prior tend to outperform the other methods, followed by the LGMR model. Within prior choices, the LGMR model tends to produce the best performance. Surprisingly, *t* test tends to strongly outperform limma-trend, which rapidly decays with significance level while *t* test peaks around 1%.

As for the Bruderer-DS, in the Navarro-DS (Supplemental Fig. S9), we found that the prior choice had the biggest impact on Baldur’s performance. In particular, the EB prior outperformed the other methods followed by GR-WI, limma-trend, *t* test, and LGMR-WI. This could be due to the trend in this DS having surprisingly flat variance for a large range of means (approx. 5–8; Supplemental Fig. S1*F*). The GR model does not capture this which could lead to inaccurate uncertainty inference, while the LGMR does (Fig. 1*F*). Still, in contrast to the other methods, both LGRM models increase in MCC over the entire range of significance levels investigated here.

In conclusion, from the MCC evaluation, we found that Baldur tends to make more balanced decisions around traditional significance levels. In addition, we found that Baldur retains a more balanced decision over a wide range of decisions compared to limma-trend and *t* test and is particularly good in comparisons where all models have their lowest performance (*i.e.*, has the best worst-case performance; except LGMR-WI in the Navarro-DS). Finally, we found that both EB and WI generally perform similarly from a balanced decision perspective. Still, the LGRM model generally shows the highest performance across all DSs and comparisons independent of prior choice.

**Fig. 4:**
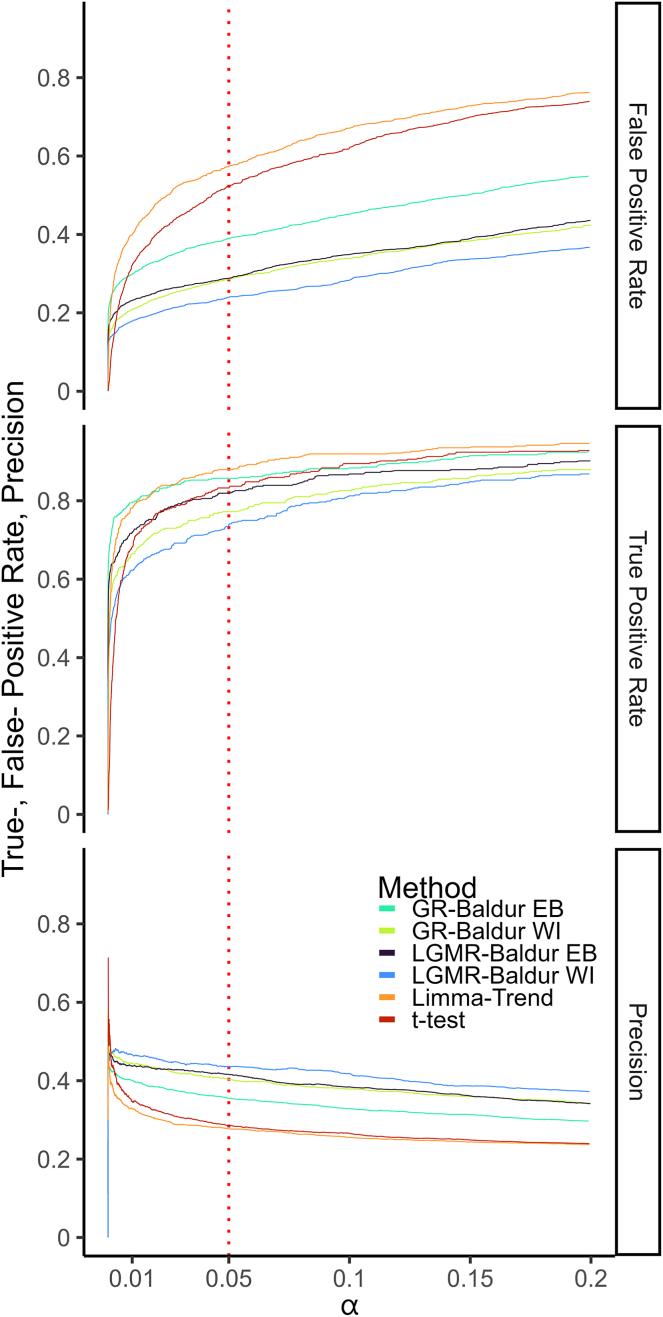
**Performance metrics of the Yeast-DS plotted against the significance level (α).***y*-axis shows the metric value (as indicated by *facet titles*), *x*-axis shows the significance level, and colors as described in Figure 2.

Next, we analyzed the TPR, the FPR, and precision as a function of the significance level. For the Yeast-DS (Fig. 4), we see an improvement, foremost in controlling the FPR, while still being competitive in TPR. While all models produce large FPR, Baldur shows robust control at small significance levels and slowly increases in FPR. On the other hand, limma-trend and *t* test rapidly pick up FPs at lower significance levels. In particular, we observe that LGMR model controls the FPR well for both priors, while the GR inference with the EB prior tends to produce the largest TPR and FPR of the Baldur methods. Importantly, the control of the FPR leads to increased precision of the Baldur methods, all improving over limma-trend and *t* test.

For the Ramus-DS (Supplemental Figs. S10 and S11), we again observed that Baldur models tend to control the FPR to a much higher degree, while having similar or even better TPR. In particular, we observed that the LGMR-EB setup vastly outperforms limma-trend in reducing FPR, while attaining marginal improvement in TPR. We observe that the improvements in Baldur methods come from better control of the FPR rather than an increase in TPR. In addition, Baldur controls the FPR robustly in all comparisons, while limma-trend and *t* test have fluctuating FPR.

Next, we investigated the performance of the pairwise statistical decisions in the UPS-DS (Supplemental Fig. S12) which had the second highest imbalance of the DSs investigated here (Table 1). We found that the LMGR had the best performance in terms of TPR, FPR control, and precision in all three comparisons. While the GR model had lower TPR, its FPR control still produced the second-best precision in all comparisons. For the nonpairwise contrasts (Supplemental Fig. S13), we observed a similar pattern in performance where LGMR had the best performance, GR had a lower TPR while still maintaining among the best precisions; similarly, limma-trend produced a significantly higher FPR. The third comparison at low spike-in log fold change (−0.32; fold change 0.8) all methods had very low TPR. Limma-trend made no decision until a large significance level of roughly 0.18, while LGMR-EB managed to produce the largest TPR among all methods.

For the Human-DS (Supplemental Fig. S14), we saw that Baldur methods have outstanding precision over limma-trend and *t* test due to the strong FPR control in this DS. Further, all models can get a high TPR, except for GR in *1:12 versus 1:6*. Still, we observed that, in the worst-case scenario, Baldur-based models make an improved decision by better controlling the FPR. Since these DSs have large TN, this translates to increased precision for all Baldur methods over limma-trend, with the WI prior slightly outperforming the EB.

In the Bruderer-DS (Supplemental Fig. S15), we found that limma-trend tends to rapidly amass FPs compared to the other methods. Limma-trend and LGMR-EB acquire TPR fastest and at similar rates, followed by LGMR-WI and *t* test, while the GR based inference tends to accumulate TPR at a slower rate. Still, the GR models and LGMR-WI tends to have the best precision, followed LGMR-EB and *t* test, though, both *t* test and limma-trend drop surprisingly fast in precision with significance level.

Finally, for the Navarro-DS (Supplemental Fig. S16), we again see that limma-trend—followed closely by *t* test—accumulates FPR faster than the Baldur models, where the GR-EB has the highest FPR rate. Similarly, the GR-EB and LGMR-EB has the highest TPR among the Baldur models and is slightly lower than limma-trend and slightly higher than *t* test. In addition, we found that the WI prior lowers TPR more than the other methods but still tends to have high precision together with the LGMR-EB. Finally, we observe that GR-EB has the lowest precision among the Baldur methods and limma-trend as well as *t* test have even lower precision.

**Fig. 5:**
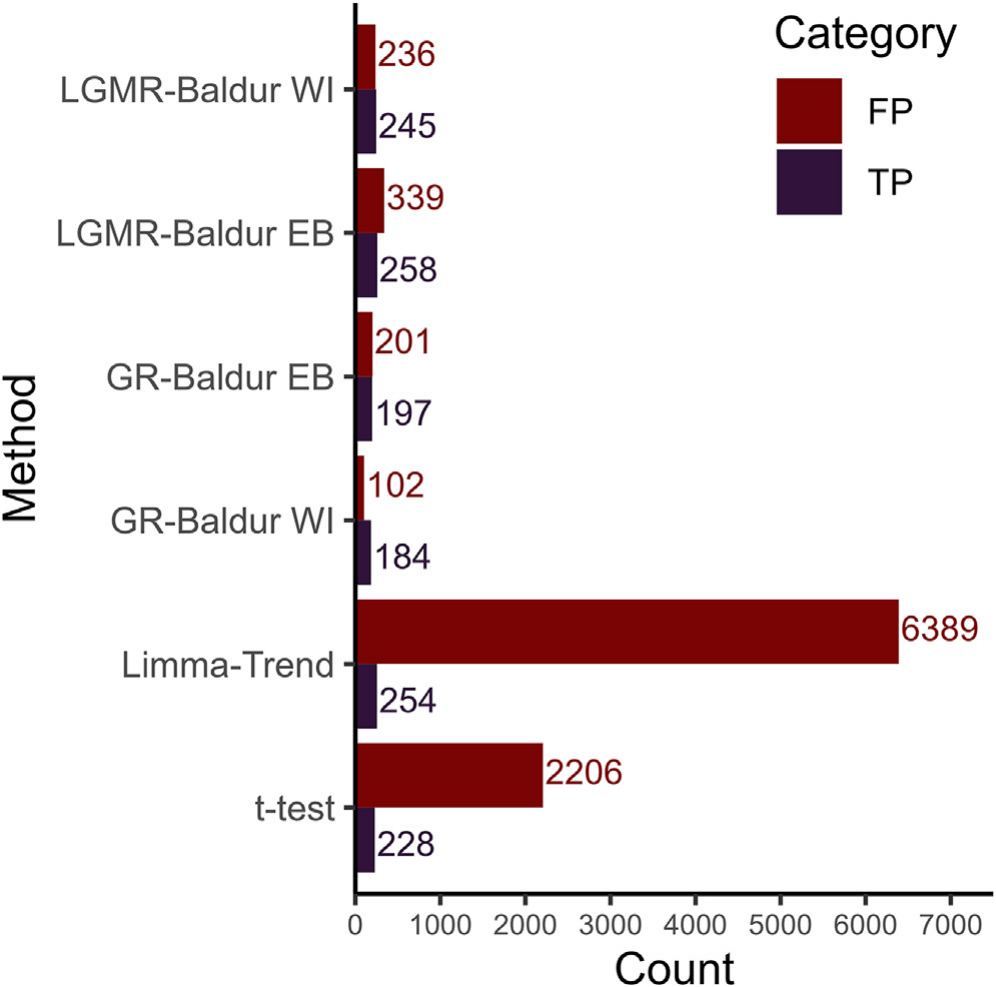
**Number of false positives and true positives of at 5% significance level for Bruderer-DS.** The *y*-axis shows the different models, and *x*-axis shows number of false positives (*red color*) or true positives (*blue color*).

Concludingly, we observed that investigating performance metrics as a function of significance level can elucidate model-specific behavior. In particular, Baldur’s methods tend to control the FPR much better than *t* test and limma-trend while generally having comparable TPR. This led to the best precision in all DS and comparisons for Baldur. Concurrently, we found that this led to an improvement in a balanced decision as measured by the MCC, indicating that Baldur’s methods tend to make more balanced decisions. This becomes obvious when examining the distribution of FP and TP at the 5% significance level for all six DSs (Fig. 5 and Supplemental Figs. S17–S21), where Baldur based methods tend to have similar TP but far lower FP. The exemption is for a few comparisons in the Ramus-DS and in the UPS-DS, where the LGMR method picks up more TPs and fewer FPs.

### Empirical Power Analysis

**Fig. 6:**
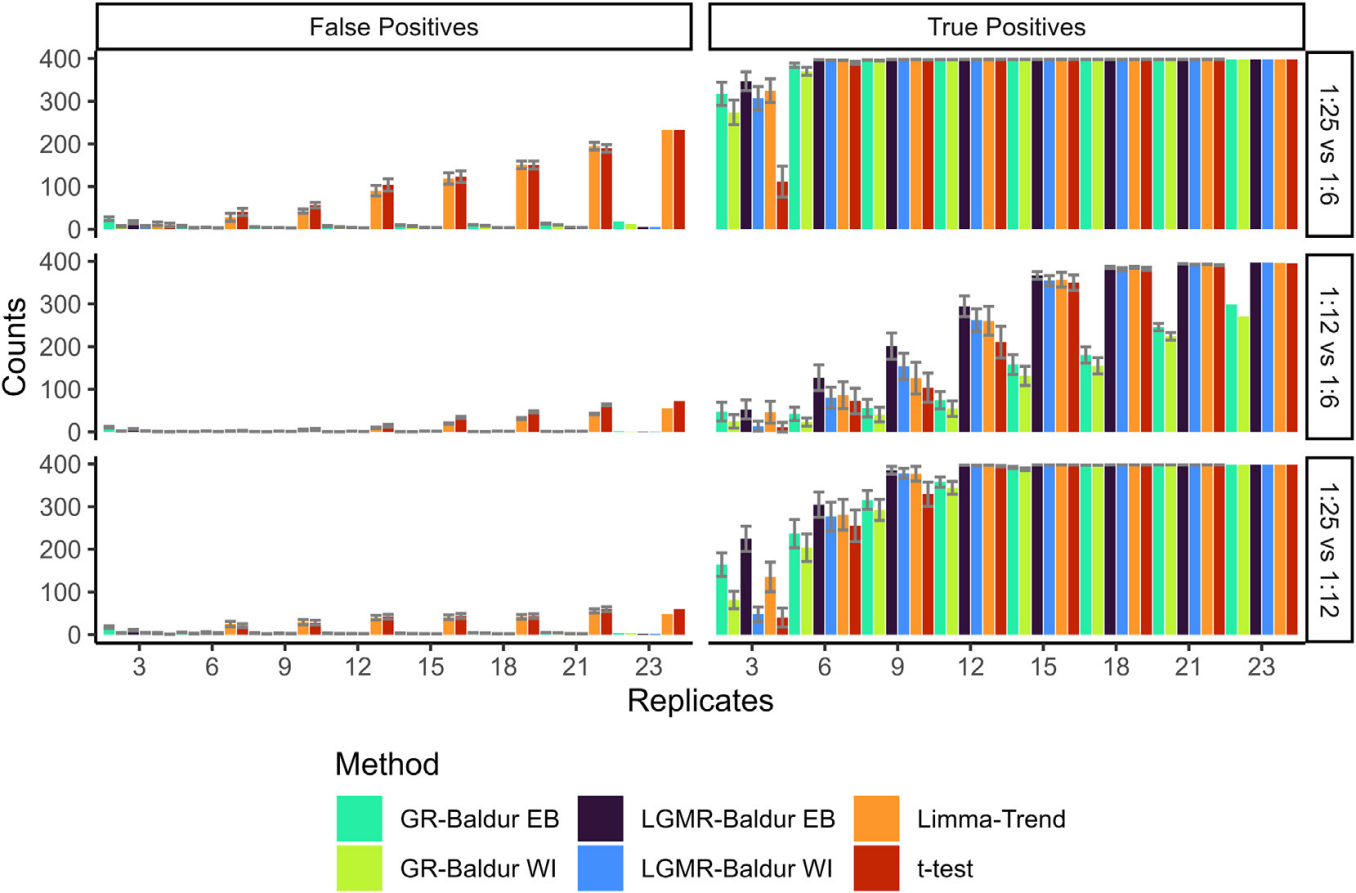
**Baldur increases in power with increasing number of replicates without the expense of increased false positives in the Human-DS.***Bars* show the average number of false or true positives (indicated by *facet titles*) at a 5% significance level of n = 24 independent runs with each run having a unique subset of replicates. Error bars shows the mean ± SEM, 24 replicates are all replicates in the data and therefore a point estimate. *x*-axis indicates the number of replicates used. Colors as described in Figure 2.

As a final analysis, we investigated the performance of the methods presented here as a function of replicates. The large number of replicates in the Human-DS (Table 1) allowed us to analyze how the number of replicates affects the statistical decision. To this end, we varied the number of replicates from 3 to 21 within each condition by producing 24 randomly selected combinations for each replicate size. We then investigated the TP and FP at a 5% significance level for each replication size and combination. For the first comparison, we found that all methods except *t* test could identify most TPs at three replicates, and all methods could identify all TPs at six replicates (Fig. 6). Surprisingly, as the number of replicates increased, both *t* test and limma-trend increased in the number of FPs. On the other hand, Baldur methods remained steadily at the same number of FPs for the entire range of replicates; this held true for all three comparisons in the DS. For the second comparison, we again observed that *t* test and limma-trend increased in FPs as the number of replicates increased but flatten around 18 to 21 replicates. In addition, we see that GR with both priors have subpar TP detection compared to the others that identified nearly all TPs around 18 replicates. Finally, we observed that in the third comparison, LGMR and limma-trend could identify more TPs than other methods at three replicates. At nine replicates, we found that all methods identified most of the TPs and that there was no change in FPs as replicates increased. Taken together, Baldur’s decision benefits from increasing the number of replicates. On the other hand, increasing the number of replicates can reduce the performance of *t* test and limma-trend by reducing control of the FPR.

## Discussion

While increasing TPR is a driver for proteomics discovery, controlling the FPR is critical for precise high-throughput functional analysis of proteomes and reactomes and enables a better downstream systems biology analysis. In addition, increased FPR control will be directly associated with improved reproducibility of a study. Here, we demonstrate that Baldur with the LGMR model has the best performance across all comparisons in all six DSs investigated independent of them, exhibiting a mixture of distributions or not (Fig. 1 and Supplemental Fig. S1).

We compared two different priors for the mean of each condition, a WI (Equation 9) and a data-driven EB (Equation 8). In general, we found comparable performance, but the WI prior tends to produce smaller TPR while having higher precision. On the other hand, the EB prior tends to produce higher TPR but with lower precision. Compared to *t* test and limma-trend, the EB prior has a comparable TPR but reduced FPR, while the WI has lower TPR but even smaller FPR. As such, if the analyst is interested in a well-annotated list of peptides with a high confidence of being TPs they should use the WI prior; if the interest is in maximizing the number of TPs at the expense of more FPs, they should use the EB prior. Further, we find that the inference of uncertanties and hyperparameters for Baldur with the LGMR model developed here outperforms GR in all comparisons, making it the *de facto* method of choice. Likely, this is due to the improved M-V trend modeling of the LGMR model being able to infer it locally for each peptide in the DSs. Importantly, the LGMR model estimation time scales linearly with the number of rows in the data (Supplemental Fig. S22*A*), while the data and decision models running time decreases exponentially with the number of parallel workers (Supplemental Fig. S22*B*) and all DS investigated here converged to similar times from 1 to 8 min with a large number of threads. For a modern laptop using ten parallel workers the typical time would be around 3 to 30 min depending on the number of peptides, conditions and comparisons in the DS. In particular, we find that the LGMR model is only impacted by the number of peptides while the data and decision model is mainly impacted by the number of conditions and comparisons, while being unaffected by the number of replicates (Table 1 and supplemental Fig. S22*A*).

In the six DSs investigated here (Supplemental Fig. S1), we found that the LGMR model ((17), (18), (19), (22)) described the M-V trends well (Fig. 1 and Table 2). Still, while the LGMR model performed well on all DSs analyzed in this study, there is no guarantee that it will fit any input DS produced by the vast number of spectral quantification methods. Importantly, using Baldur with a GR model still tends to outperform *t* test and limma-trend and pose as a valid option for the LGRM at a lower computational cost.

Lastly, we believe that Baldur’s robust control of false alarms will be highly effective for analyzing PTM and peptidomics DSs susceptible to have larger sample variations. Here, we observed this in Yeast-DS (PTM DS), Ramus-DS for small spike-in concentrations, and in the Bruderer total proteomics DS, where Baldur’s FP control was superior compared to *t* test and limma-trend.

## Dedication

The authors would like to dedicate this paper to the memory of Professor Sorina Popescu.

## Data Availability

Baldur is available through GitHub (https://github.com/PhilipBerg/baldur) and through The Comprehensive R Archive Network (**install.package(”baldur”)**). Yeast-DS, UPS-DS, and Ramus-DS (PRIDE IDs PXD009694, PXD009693, PXD001819, respectively) quantifications are available through the figshare repository (https://figshare.com/s/28e837bfe865e8f13479); Yeast-DS as well as UPS-DS are included in the Baldur package. The Human-DS and Bruderer-DS are available through the original publications (20, 21, European Genome-phenome Archive, https://ega-archive.org, accession EGAD00010002223; PeptideAtlas http://www.peptideatlas.org, No. PASS00589, respectively). The Navarro-DS was downloaded from MassIVE.quant (48, ID RMSV000000250.2). The R code used to produce this paper is available through GitHub (https://github.com/PhilipBerg/baldur_code).

## Supplemental data

This article contains supplemental data.

## Conflict of interest

The authors declare that they have no competing interests.
