# Supplementary material for: Baldur: Bayesian Hierarchical Modeling for Label-Free Proteomics with Gamma Regressing Mean-Variance Trends
Source: Mol Cell Proteomics. 2023 Oct 7;22(12):100658. doi: 10.1016/j.mcpro.2023.100658 (PMC10687340; doi:10.1016/j.mcpro.2023.100658)
Supplement: Supplemental Data [file mmc1.pdf]

Supplementary Figures

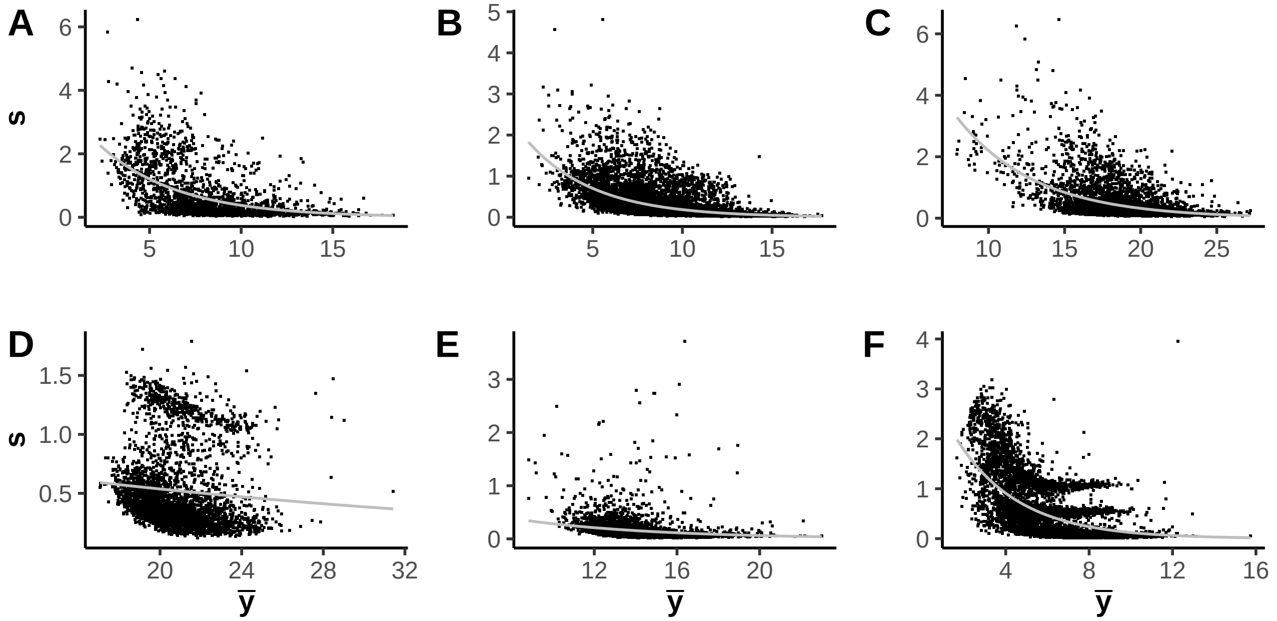

Figure S1: Mean-variance trend in the Yeast-, UPS-, Ramus-, and Human-DS (**A**-**D**, respectively) with the estimated gamma regression. X-axis shows the sample mean, Y-axis shows the sample standard deviation, and the gray line represents the estimated gamma regression model.

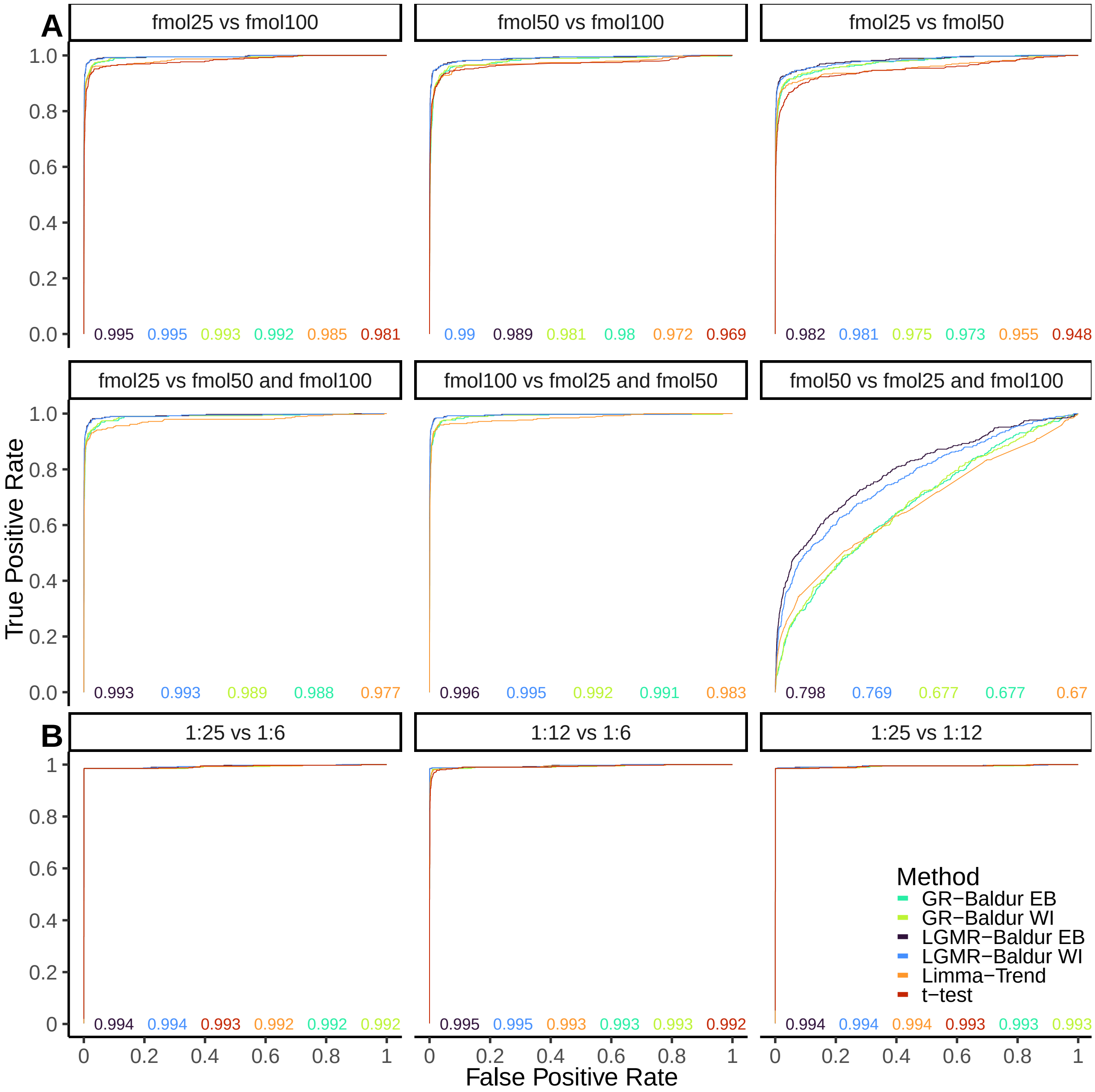

Figure S2: Receiver operator characteristic curves for the three comparisons in the UPS-DS (**A**) and Human-DS (**B**). Facet titles indicate what comparison the plot graphs. For the UPS-DS, titles show the spike-in concentrations of UPS1 (25-, 50-, or 100-fmol; 1:2:4), and, for the Human-DS, they show the human to *E. coli* peptide ratio (i.e., human:*E. coli*). "and" indicates the mean of the two conditions (e.g., contrast vector [-1 0.5 0.5]^{T}). The X-axis shows the false positive rate, and the Y-axis shows the true positive rate.

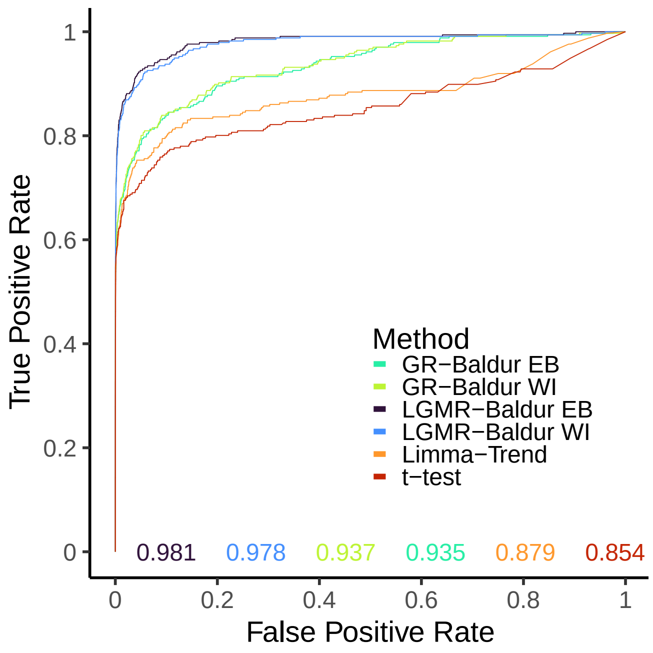

Figure S3: Receiver operator characteristic curves for the Bruderer-DS. The X-axis shows the false positive rate, and the Y-axis shows the true positive rate.

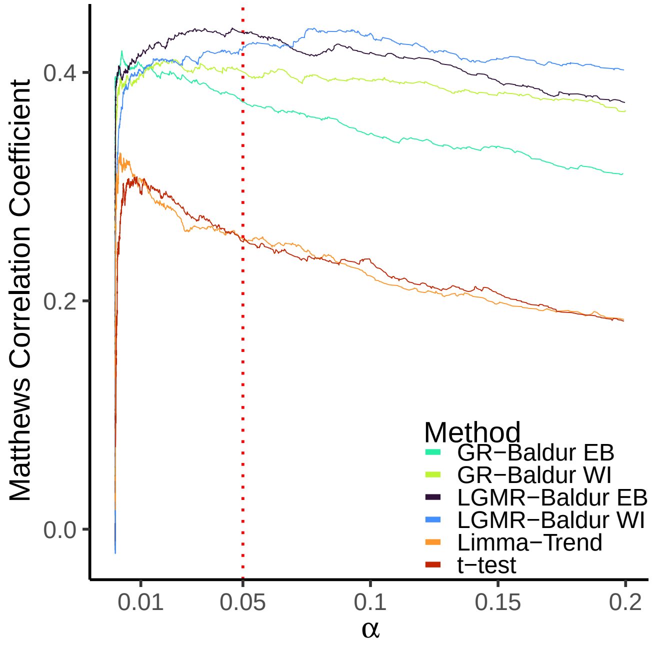

Figure S4: Mathews correlation coefficient of the Yeast-DS plotted against the significance level (α). Y-axis shows the Mathews correlation coefficient, and the X-axis shows the significance level.

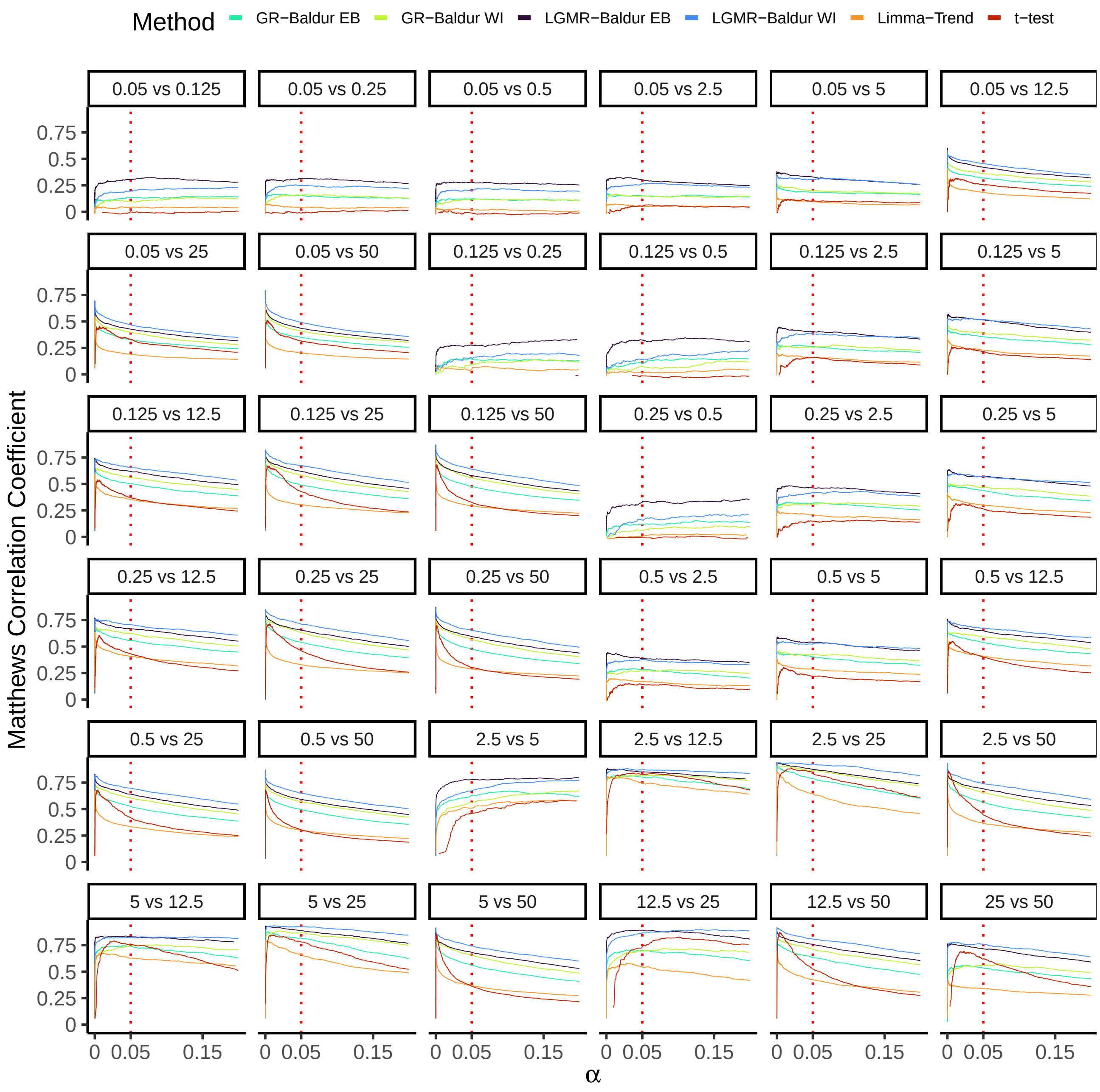

Figure S5: Mathews correlation coefficient of the Ramus-DS plotted against the significance level (α). Y-axis shows the Mathews correlation coefficient, and the X-axis shows the significance level for the different comparisons (as indicated by the X-axis facet titles).

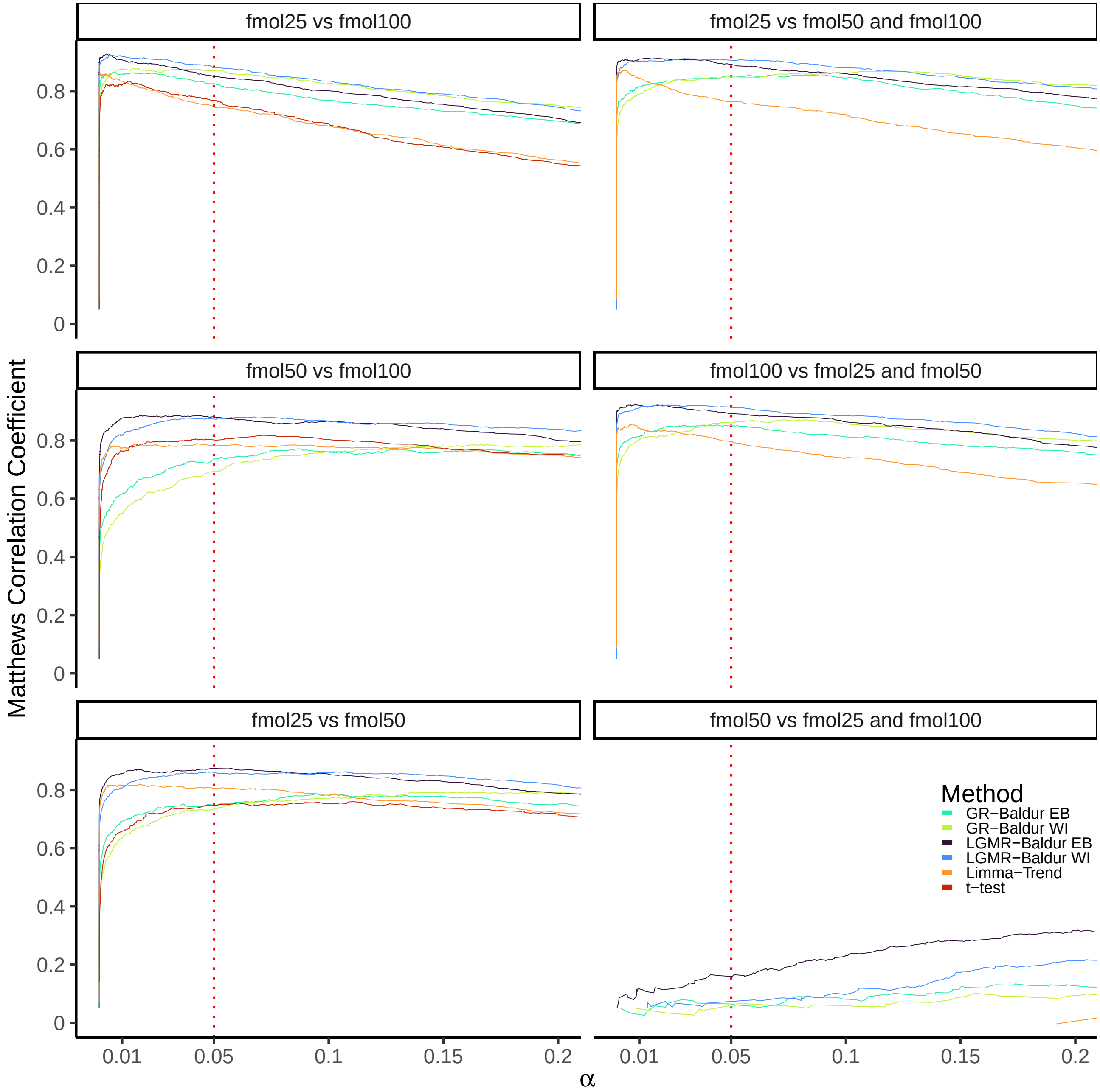

Figure S6: Mathews correlation coefficient of the UPS-DS plotted against the significance level (α). Y-axis shows the Mathews correlation coefficient, and the X-axis shows the significance level for the different comparisons (as indicated by the X-axis facet titles).

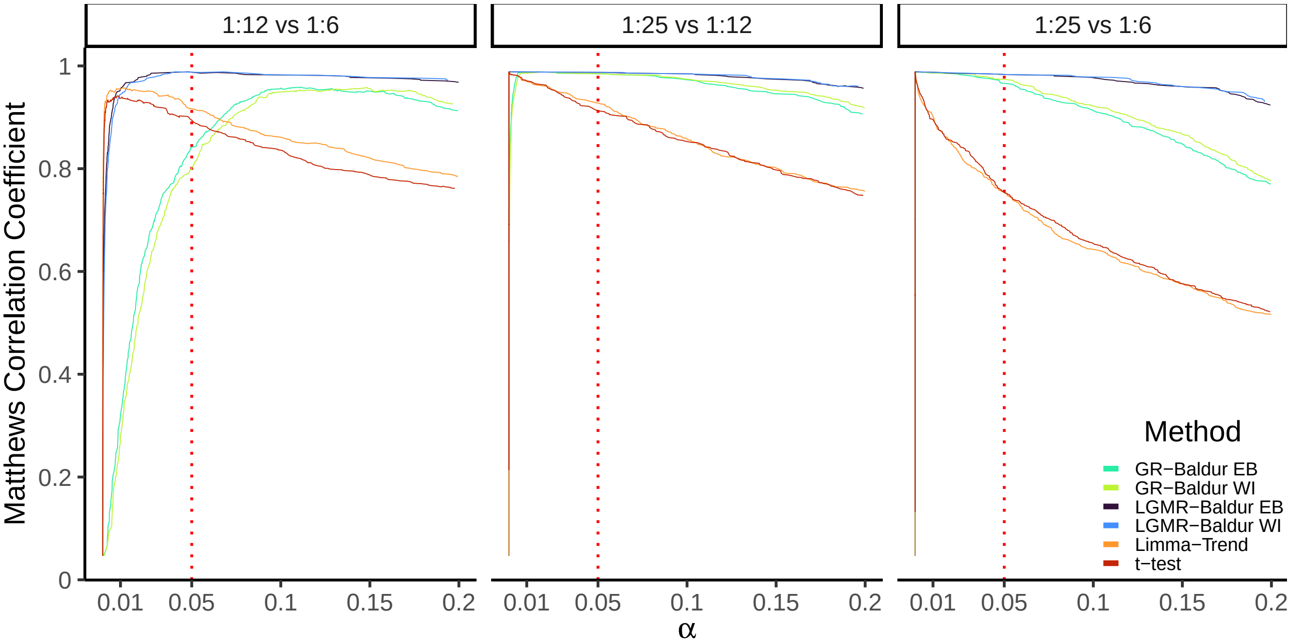

Figure S7: Mathews correlation coefficient of the Human-DS plotted against the significance level (α). Y-axis shows the Mathews correlation coefficient, and the X-axis shows the significance level for the different comparisons (as indicated by the X-axis facet titles).

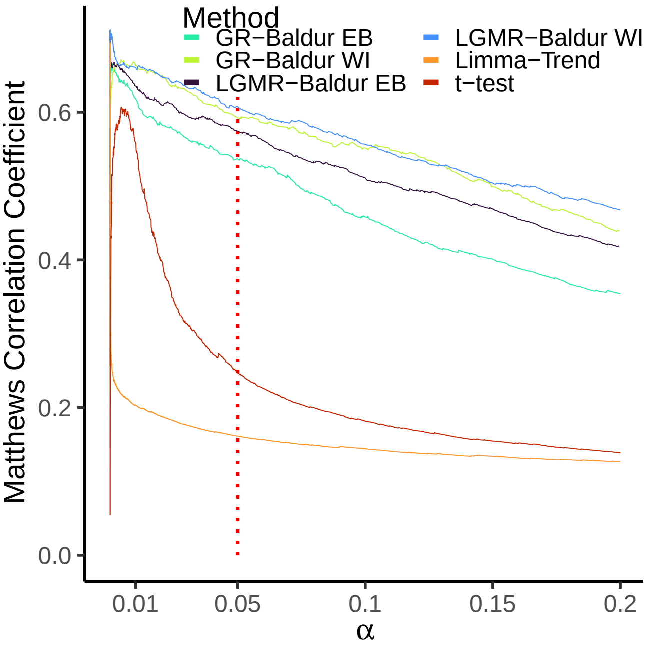

Figure S8: Mathews correlation coefficient of the Bruderer-DS plotted against the significance level (α). Y-axis shows the Mathews correlation coefficient, and the X-axis shows the significance level.

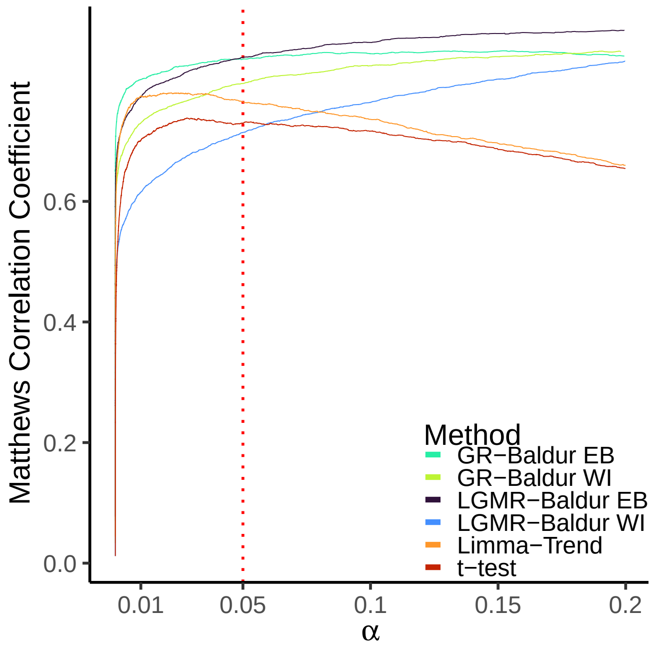

Figure S9: Mathews correlation coefficient of the Navarro-DS plotted against the significance level (α). Y-axis shows the Mathews correlation coefficient, and the X-axis shows the significance level.

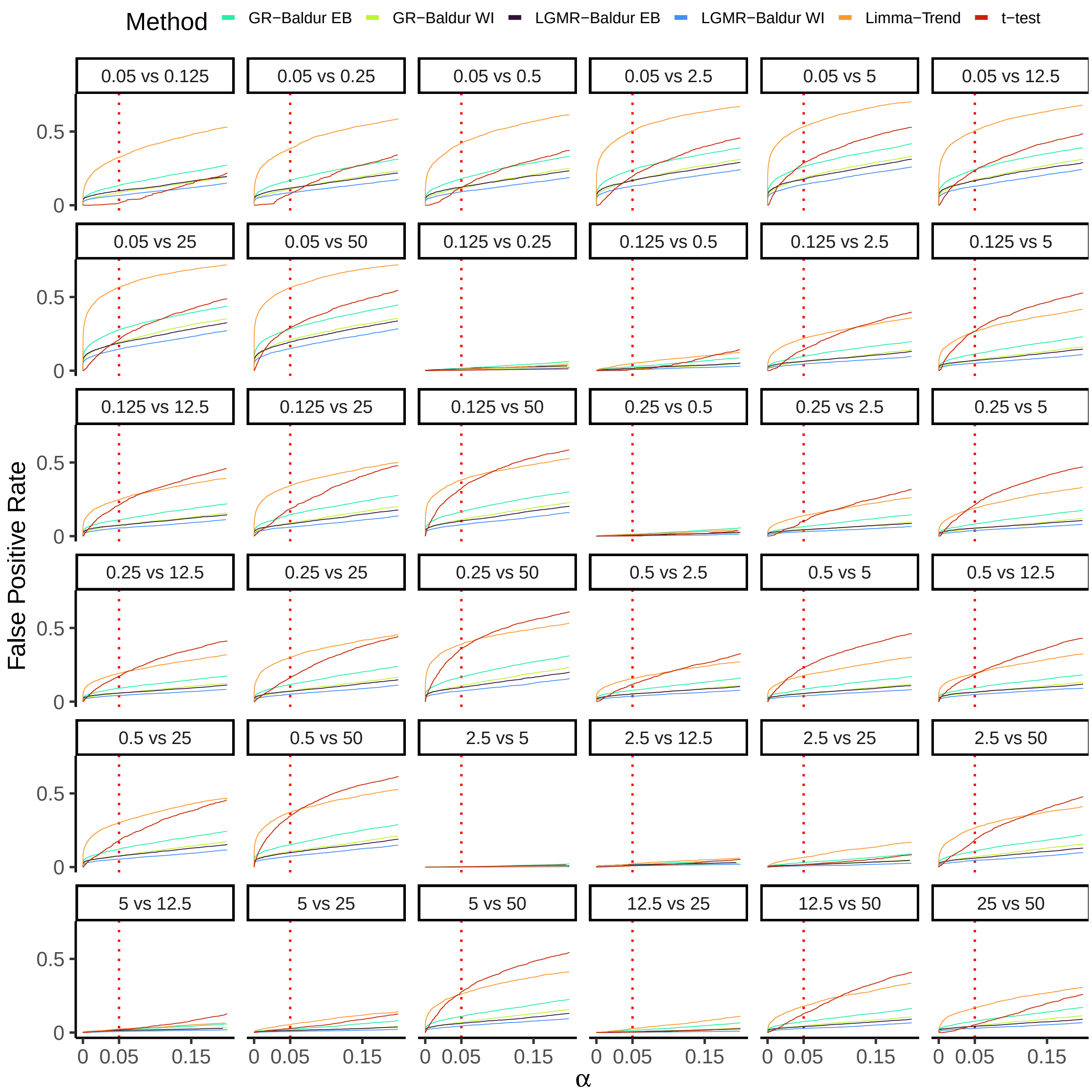

Figure S10: False positive rates of the Ramus-DS plotted against the significance level (α). Y-axis shows the false positive rate, X-axis shows the significance level for the different comparisons (as indicated by the facet titles).

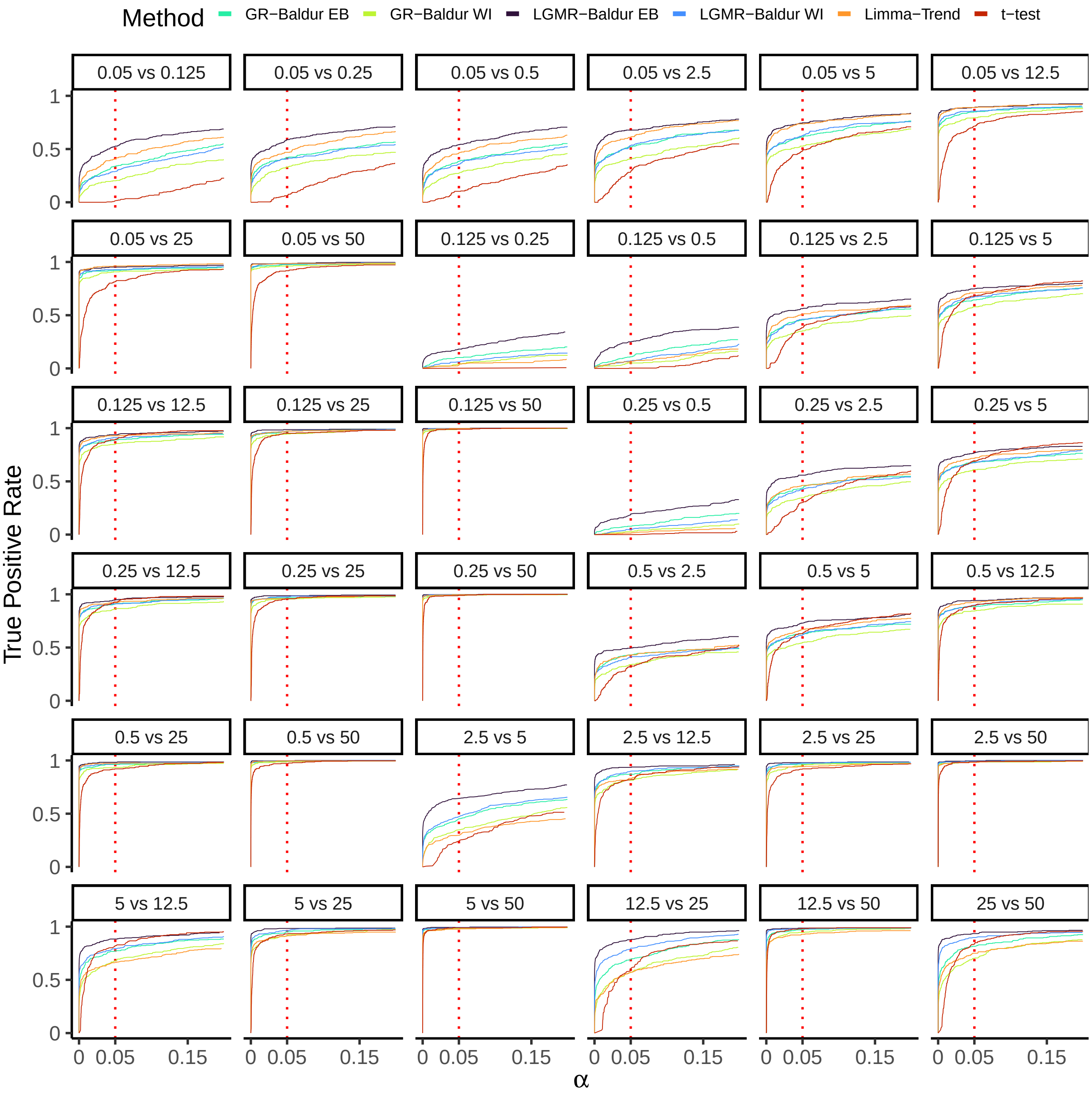

Figure S11: True positive rates of the Ramus-DS plotted against the significance level (α). Y-axis shows the true positive rate, X-axis shows the significance level for the different comparisons (as indicated by the facet titles).

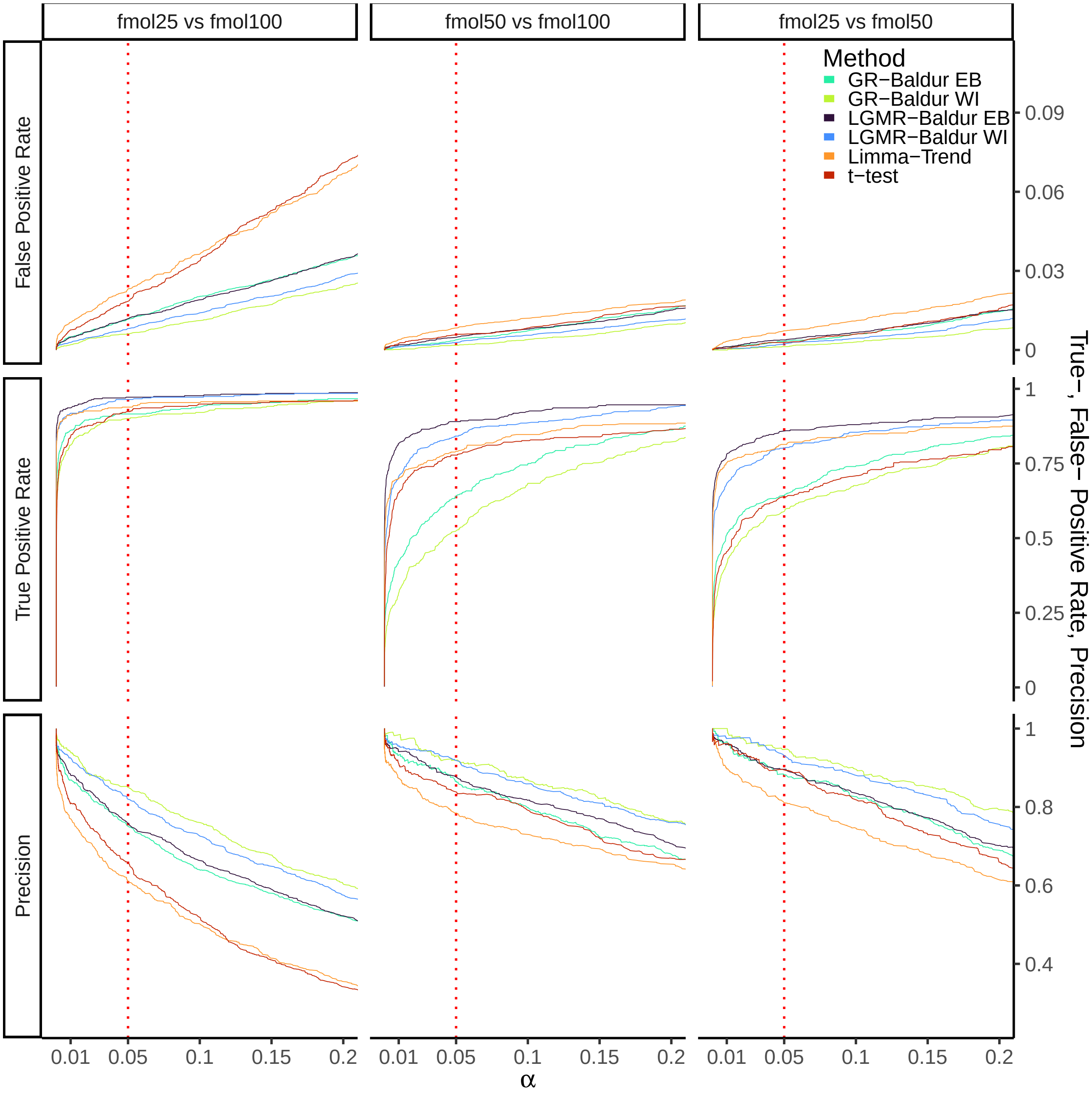

Figure S12: Performance metrics of the pairwise comparisons in the UPS-DS plotted against the significance level (α). Y-axis shows the metric value (as indicated by the Y-axis facet titles), and the X-axis shows the significance level for the different comparisons (as indicated by the X-axis facet titles).

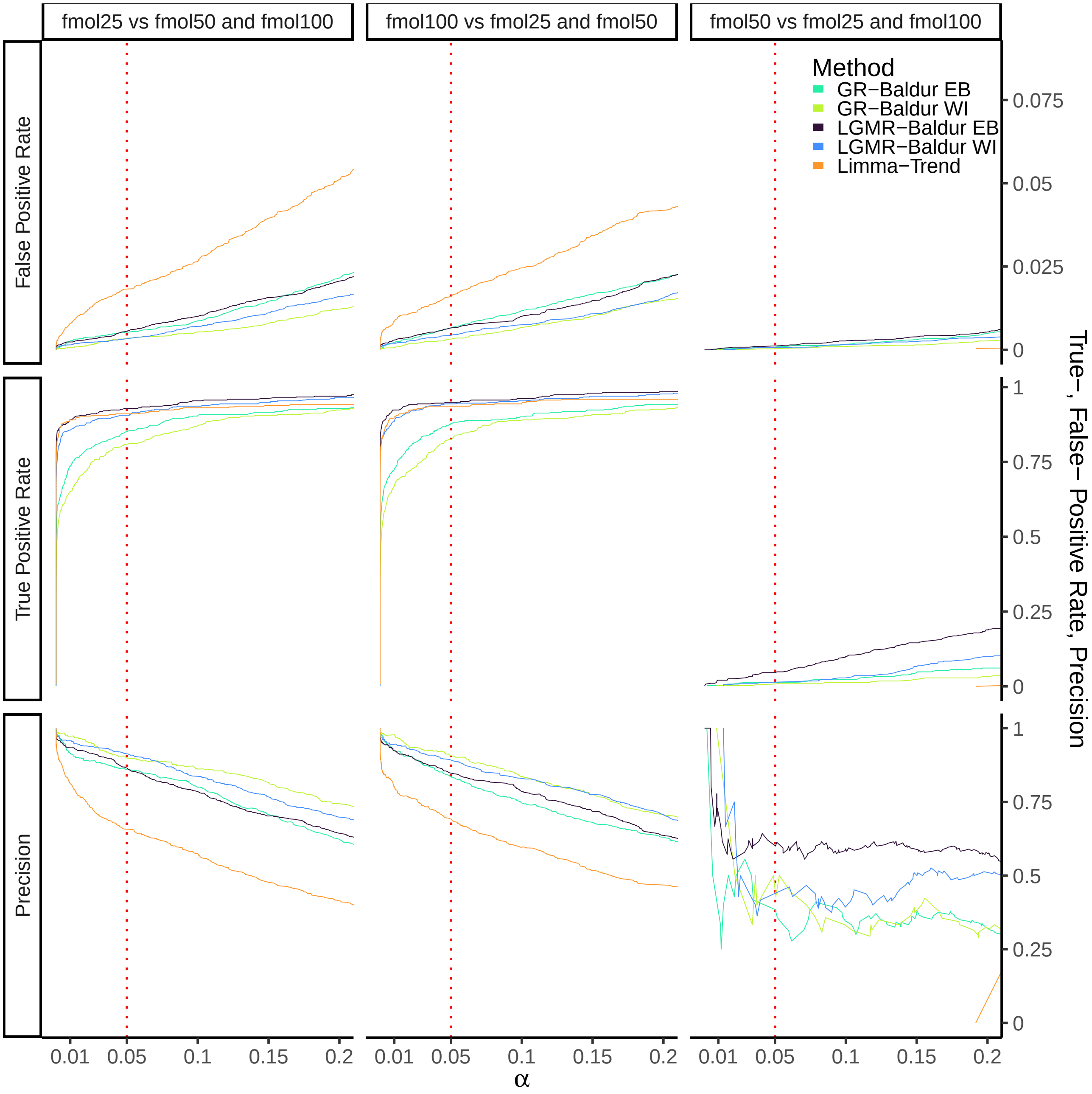

Figure S13: Performance metrics of the complex contrasts in the UPS-DS plotted against the significance level (α). Y-axis shows the metric value (as indicated by the Y-axis facet titles), and the X-axis shows the significance level for the different comparisons (as indicated by the X-axis facet titles). "and" indicates the mean of the two conditions (e.g., contrast vector [-1 0.5 0.5]^{T}).

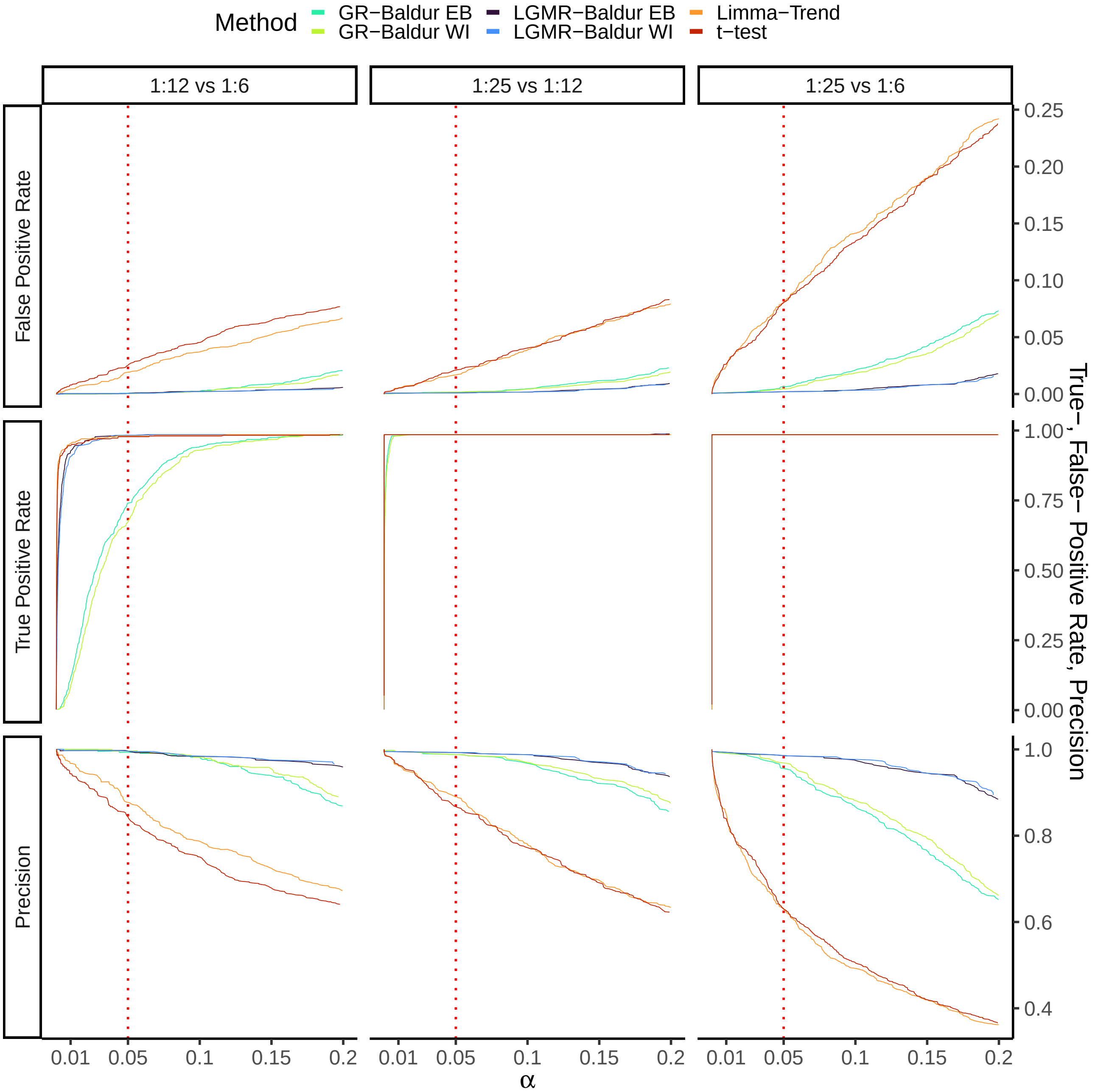

Figure S14: Performance metrics of the Human-DS plotted against the significance level (α). Y-axis shows the metric value (as indicated by the Y-axis facet titles), and the X-axis shows the significance level for the different comparisons (as indicated by the X-axis facet titles).

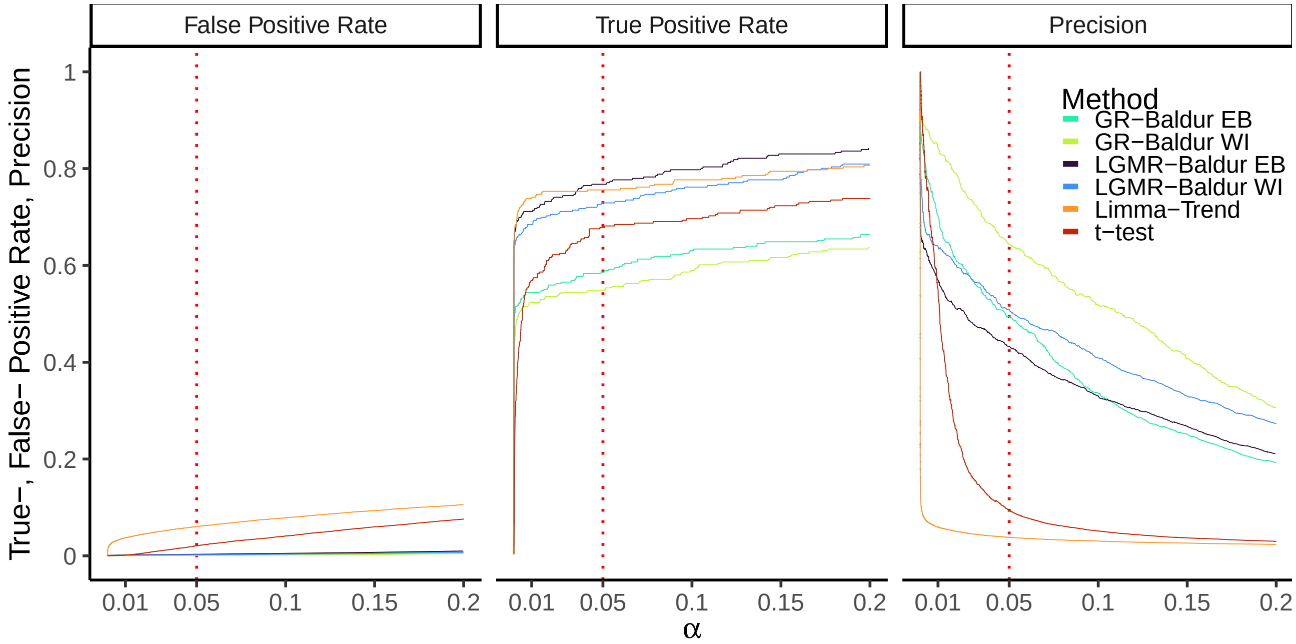

Figure S15: Performance metrics of the Bruderer-DS plotted against the significance level (α). Y-axis shows the metric value (as indicated by facet titles), and the X-axis shows the significance level.

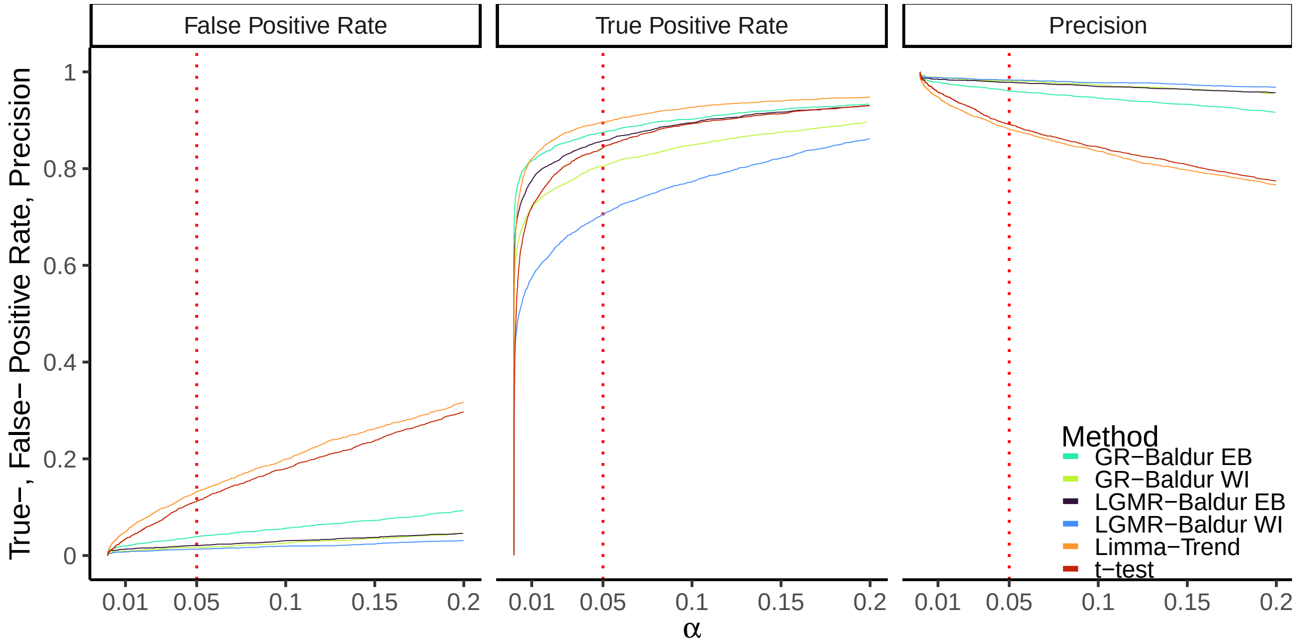

Figure S16: Performance metrics of the Navarro-DS plotted against the significance level (α). Y-axis shows the metric value (as indicated by facet titles), and the X-axis shows the significance level.

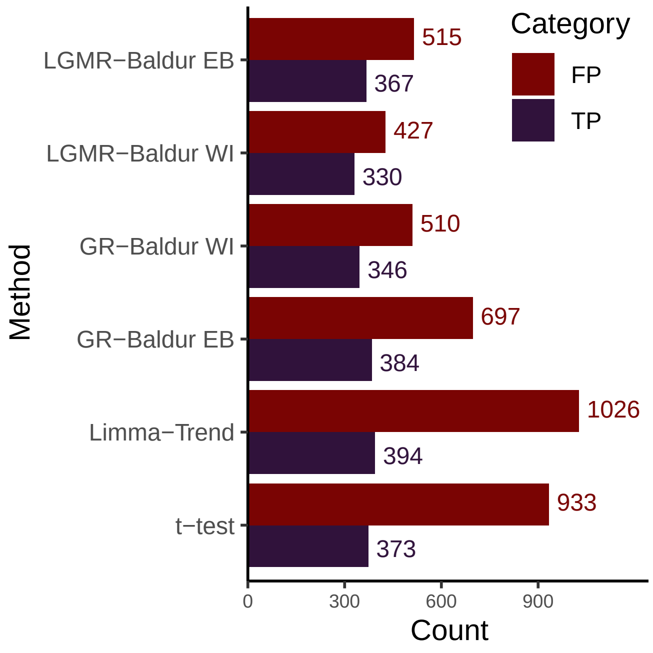

Figure S17: Decision of False Positives and True Positives of at 5 % significance level for the Yeast-D. Y-axis shows the different methods, and the X-axis shows the number of false positives (red color) or true positives (blue color).

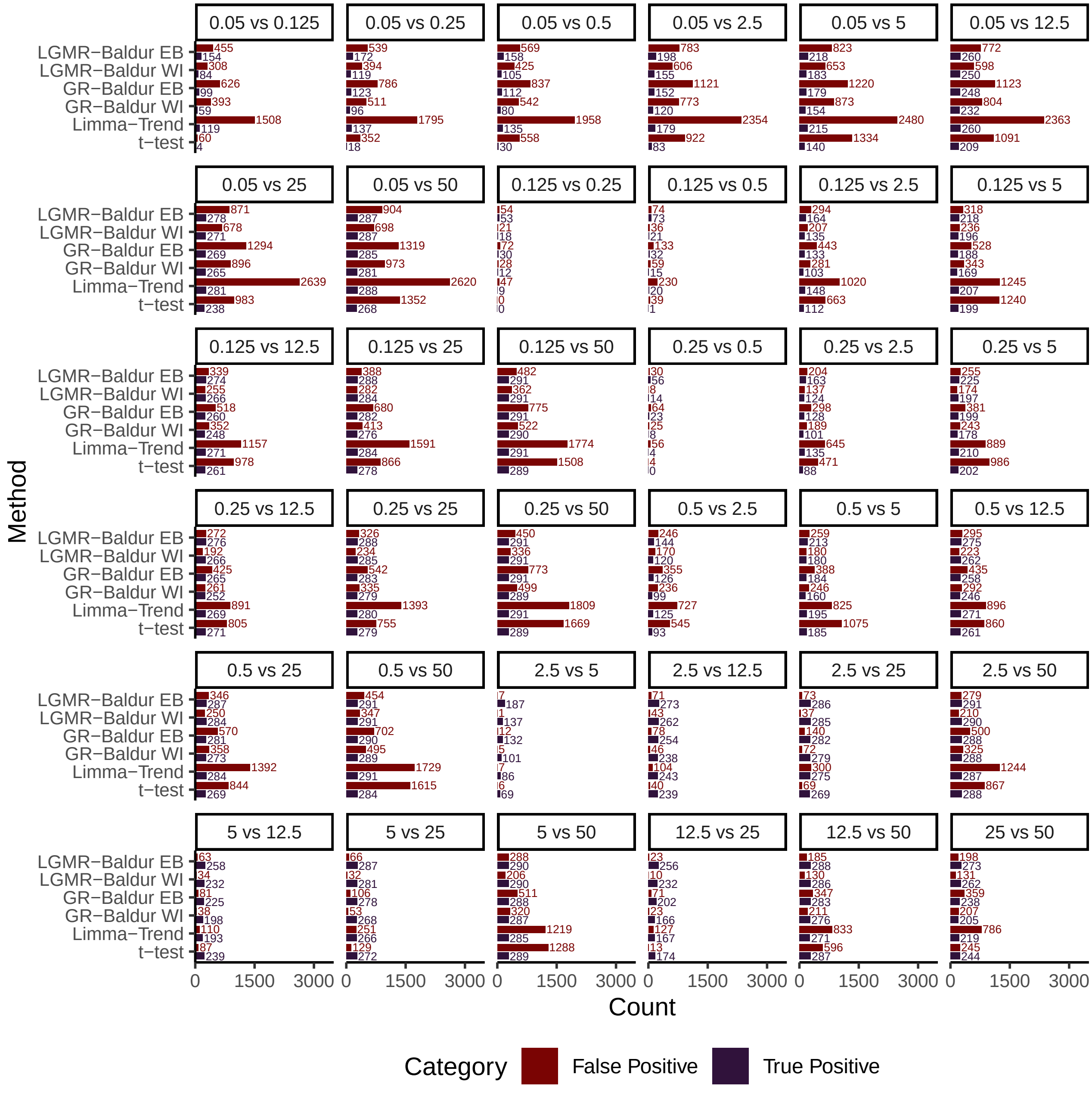

Figure S18: Decision of False Positives and True Positives of at 5 % significance level for the Ramus-DS. Y-axis shows the different methods, and the X-axis shows the number of false positives (red color) or true positives (blue color), and the facet titles indicating the comparison.

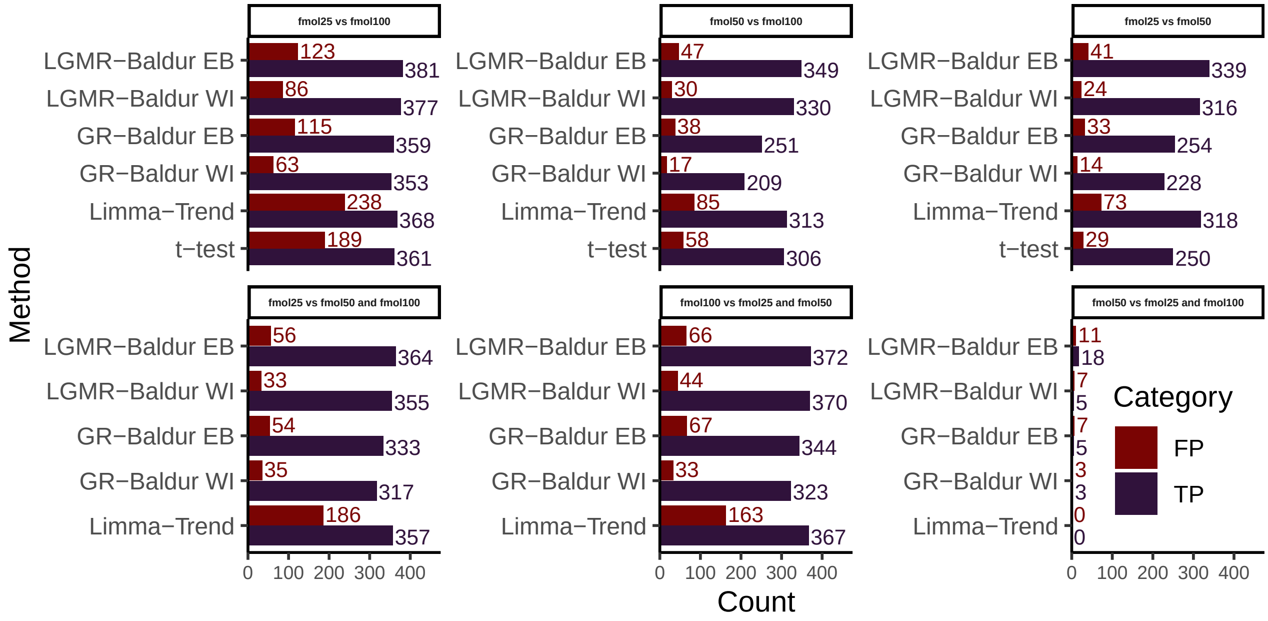

Figure S19: Decision of False Positives and True Positives of at 5 % significance level for the UPS-DS. Y-axis shows the different methods, and the X-axis shows the number of false positives (red color) or true positives (blue color), and the facet titles indicating the comparison and "x and y" implies the mean of the two conditions x and y.

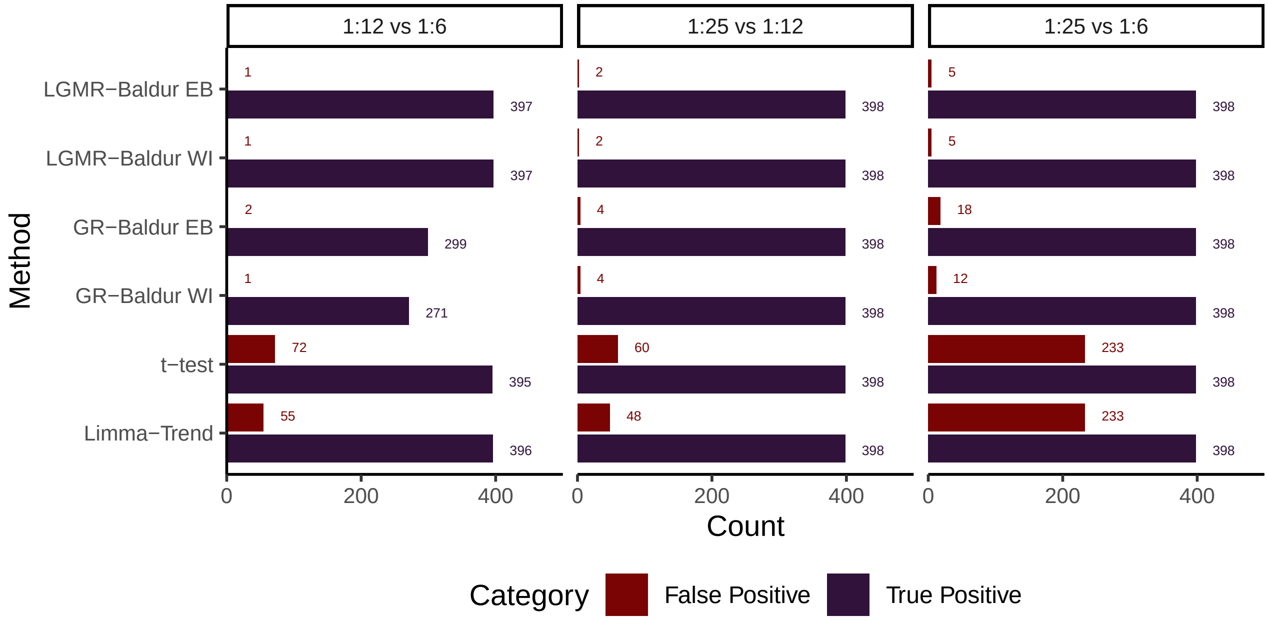

Figure S20: Decision of False Positives and True Positives of at 5 % significance level for the Human-DS. Y-axis shows the different methods, and the X-axis shows the number of false positives (red color) or true positives (blue color), and the facet titles indicating the comparison.

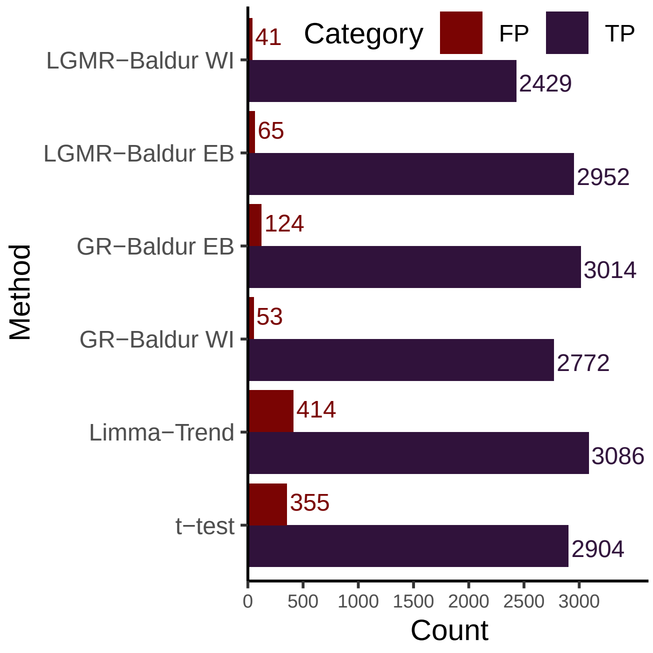

Figure S21: Decision of False Positives and True Positives of at 5 % significance level for the Navarro-DS and methods evaluated here. Y-axis shows the different methods, and the X-axis shows the number of false positives (red color) or true positives (blue color).

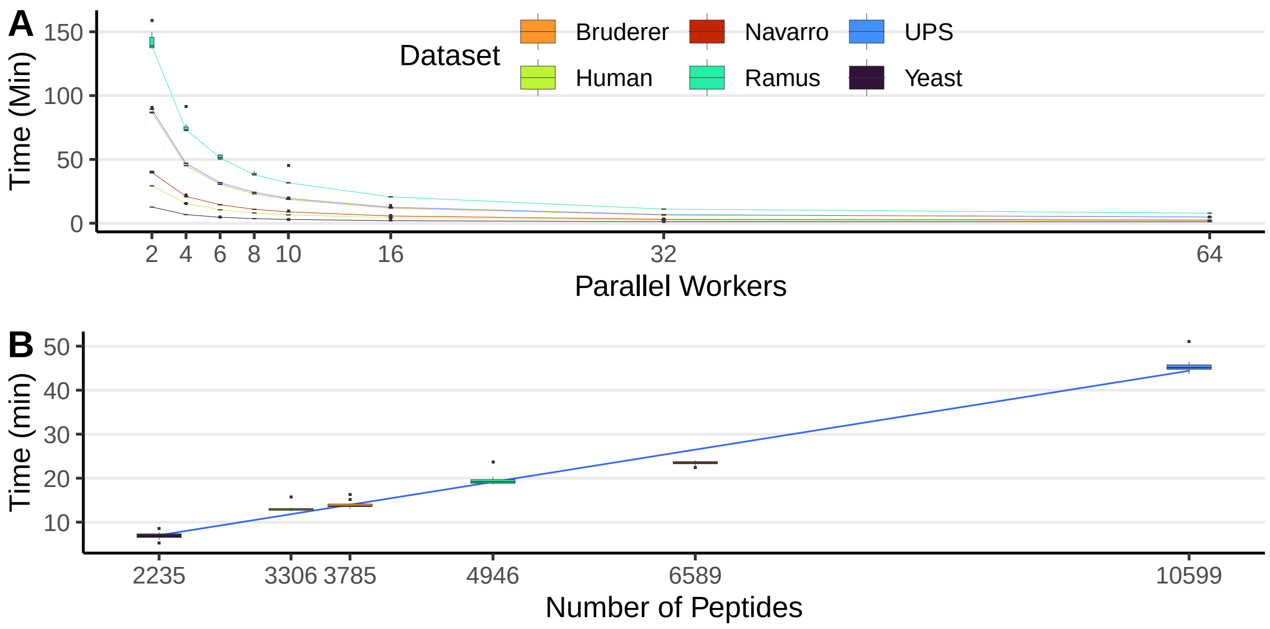

Figure S22: Box and line plot of the empirical time complexity evaluated for the Baldur models. The data and decision model (**A**) was evaluated over all investigated datasets and shows a exponential drop in time with the number of parallel workers—all converging to similar running time with large enough number of parallel workers. The LGMR model (**B**) shows a linear increase in time as the number of peptides in the dataset grows. The y-axis shows the running time in minutes and the x-axis shows the number of parallel workers (**A**) or number of peptides in the dataset (**B**). Lines show linear interpolation (**A**) or a simple linear regression (**B**).
